# OsMADS23 phosphorylated by SAPK9 confers drought and salt tolerance by regulating ABA biosynthesis in rice

**DOI:** 10.1371/journal.pgen.1009699

**Published:** 2021-08-03

**Authors:** Xingxing Li, Bo Yu, Qi Wu, Qian Min, Rongfeng Zeng, Zizhao Xie, Junli Huang

**Affiliations:** Key Laboratory of Biorheological Science and Technology of Ministry of Education, Bioengineering College of Chongqing University, Chongqing, China; The University of North Carolina at Chapel Hill, UNITED STATES

## Abstract

Some of MADS-box transcription factors (TFs) have been shown to play essential roles in the adaptation of plant to abiotic stress. Still, the mechanisms that MADS-box proteins regulate plant stress response are not fully understood. Here, a stress-responsive MADS-box TF OsMADS23 from rice conferring the osmotic stress tolerance in plants is reported. Overexpression of *OsMADS23* remarkably enhanced, but knockout of the gene greatly reduced the drought and salt tolerance in rice plants. Further, OsMADS23 was shown to promote the biosynthesis of endogenous ABA and proline by activating the transcription of target genes *OsNCED2*, *OsNCED3*, *OsNCED4* and *OsP5CR* that are key components for ABA and proline biosynthesis, respectively. Then, the convincing evidence showed that the *OsNCED2*-knockout mutants had lower ABA levels and exhibited higher sensitivity to drought and oxidative stress than wild type, which is similar to *osmads23* mutant. Interestingly, the SnRK2-type protein kinase SAPK9 was found to physically interact with and phosphorylate OsMADS23, and thus increase its stability and transcriptional activity. Furthermore, the activation of OsMADS23 by SAPK9-mediated phosphorylation is dependent on ABA in plants. Collectively, these findings establish a mechanism that OsMADS23 functions as a positive regulator in response to osmotic stress by regulating ABA biosynthesis, and provide a new strategy for improving drought and salt tolerance in rice.

## Author summary

OsMADS23 functions as a positive regulator in response to osmotic stress via ABA signaling. OsMADS23 directly activates the transcription of target genes *OsNCED2*, *OsNCED3*, *OsNCED4* and *OsP5CR* that are key components for ABA and proline biosynthesis. More importantly, the SnRK2-type protein kinase SAPK9 physically interacts with and phosphorylates OsMADS23, and increase its stability and transcriptional activity, in an ABA-dependent manner.

## Introduction

Plants are often exposed to various environmental stresses, and drought and high salinity are major stress factors that impair plant growth and productivity of crops [1]. Environmental challenges activate a complex signaling network in plants, which determine plants to achieve optimal adaption to these unfavorable stress conditions ultimately [2,3]. The adaptation response is accomplished via regulating gene expression that alters plant metabolism and growth [4,5]. Particularly, osmotic stress due to drought or salinity triggers the biosynthesis of the phytohormone abscisic acid (ABA) which, in turn, regulates a range of plant physiological processes in response to various abiotic stresses [3]. It is widely accepted that ABA binding to PYR/PYL/RCAR proteins leads to deactivation of PP2Cs, which releases and activates SnRK2 kinases [6,7]. Activated SnRK2s further pass the signals to AREB/ABF TFs through protein phosphorylation on their conserved motifs like R-X-X-S/T (where X means any amino acids) [8], thus promoting the activity of downstream TFs to modulate the expression of various ABA-responsive genes [9–11]. In Arabidopsis, SnRK2.2, SnRK2.3 and SnRK2.6 have been shown to play essential roles in regulating ABA signaling [12]. In rice, there are 10 SnRK2s (designated as SAPK1-10, osmotic stress/ABA-activated protein kinase 1–10) that are found to be activated by osmotic stress, and only SAPK8, SAPK9 and SAPK10 are activated by ABA, suggesting their functions in osmotic stress and ABA signaling [13]. As homologs of SnRK2.2, SnRK2.3 and SnRK2.6 of Arabidopsis, SAPK8, SAPK9, and SAPK10 are able to phosphorylate and activate the downstream ABRE TFs [14]. In other studies, SAPK9 is shown to activate OsbZIP46 by phosphorylation under ABA or drought stress treatment in rice [15,16]. It is demonstrated SAPK10 phosphorylates TRAB1 and OsbZIP77 *in vitro* [14,17]. Actually, SAPK6 is found to be able to phosphorylate OsbZIP46, responding to ABA signaling *in vivo* [15,16]. More interestingly, SAPK2 is also found to be able to activate OsbZIP23 and OsbZIP46 by phosphorylation and promote the transcription of a large number of genes with functions in stress responses [15,16,18], which further expands our understanding on the functions of SAPKs in ABA signaling.

It is well described that 9-*cis*-epoxycarotenoid dioxygenase (NCED) is the key rate-limiting enzyme in ABA biosynthesis in higher plants, and its activity affects ABA accumulation [19–23]. Currently, increasing evidence has demonstrated that the enhanced expression of *NCEDs* could promote ABA biosynthesis, and therefore confers the abiotic stress tolerance [24–26]. In contrast, *nced* mutants exhibit reduced ABA accumulation, repressed seed dormancy as well as abiotic stress-sensitive phenotypes to harmful environmental conditions [27,28], which is similar to that of *aba* mutants [29,30]. The first identified *NCED* gene from maize, *VP14*, is shown to be responsible for promoting seed dormancy and water stress resistance by controlling ABA levels in plants, and *vp14* mutant displays early seed germination, reduced ABA biosynthesis and elevated water loss of detached leaves [21]. In Arabidopsis, *nced3*, a loss-of-function mutant, exhibits a water deficiency-sensitive phenotype [28], and *nced3nced5* double mutant with much less ABA content displays more serious wilting phenotype than single mutant under drought stress [27]. In another research, introduction of *PvNCED1* into *Nicotiana plumbaginifolia* increases ABA levels and enhances drought tolerance [24], and *MhNCED3* into Arabidopsis results in enhanced tolerance to osmotic and cadmium stresses [26]. Recent reports have demonstrated that overexpression of *OsNCED3* in rice enhances ABA accumulation, and therefore increases drought tolerance; however, the loss-of-function mutant *osnced3* exhibits increased sensitivity to osmotic stress, accompanied by reduced ABA levels and increased stomata aperture under water stress [25,31].

MADS-box TFs have been well documented to play diverse roles in plant growth and development [32,33]. In recent years, MADS-box proteins have been shown to function as key regulators in various environmental stress responses [34–37]. However, the mechanisms that MADS-box proteins regulate plant response to abiotic stress have just begun to be revealed. In this study, we explored the roles of OsMADS23 as a positive regulator in response to drought and salt stress. Then, we found that OsMADS23 directly targets *OsNCED2*, *OsNCED3*, and *OsNCED4* to enhance ABA biosynthesis, and the knockout mutants of *OsNCED2* exhibit increased sensitivity to oxidative stress. More importantly, our results indicated that SAPK9, an upstream protein kinase, phosphorylates OsMADS23 and increases its stability and transcriptional activity in plants, in an ABA*-*dependent manner. These results reveal a regulatory mechanism that how OsMADS23 regulates plant response to osmotic stress through ABA signaling pathway. The data also help us to dissect the components on stress-responsive pathways and provide new insights, leading to novel strategies for the improvement of drought and salt tolerance in agricultural and economic crops.

## Results

### Performance of *osmads23* mutant and *OsMADS23*-overexpressing plants in growth

**Fig 1:**
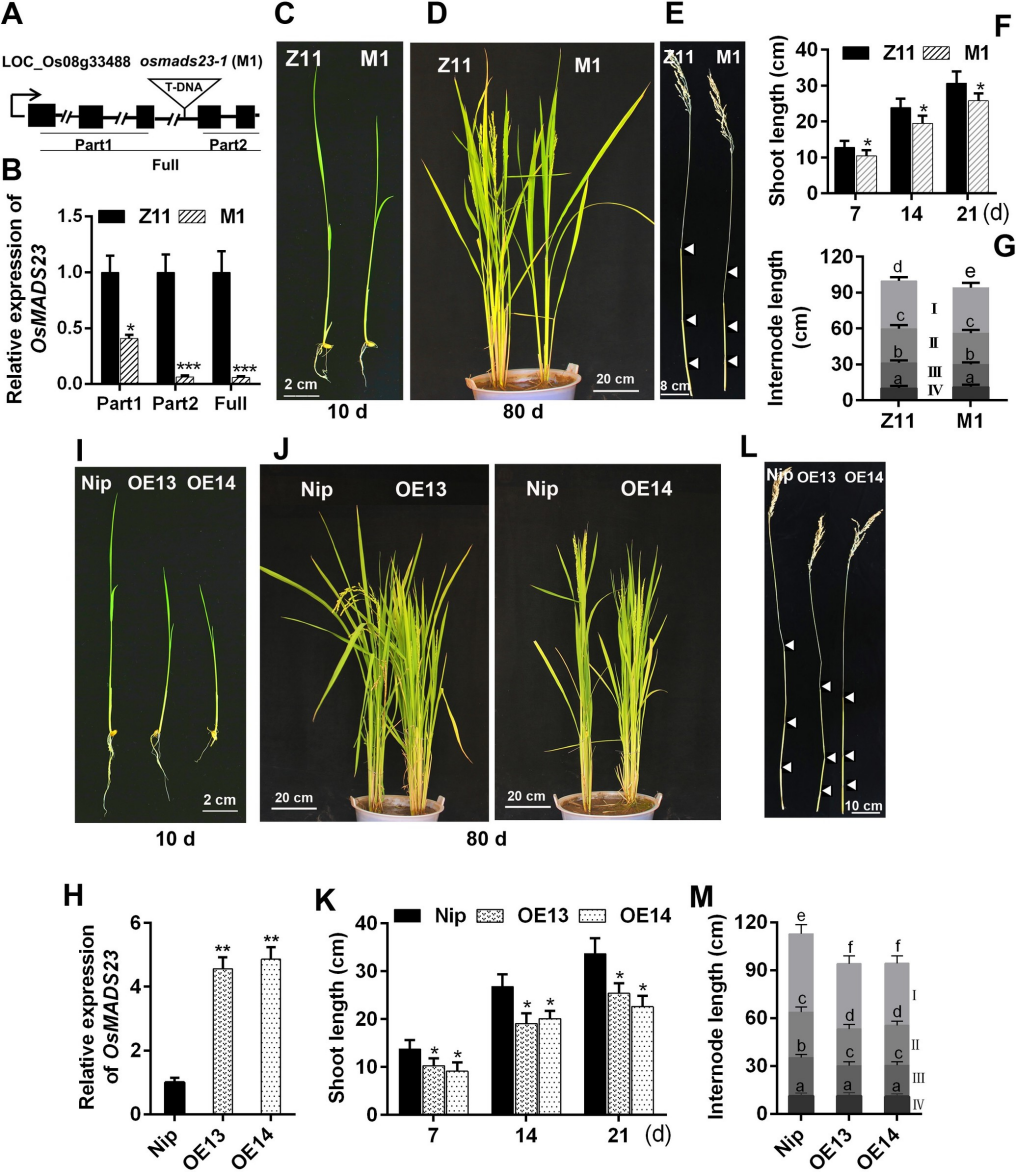
Morphological phenotypes of *osmads23* mutant and *OsMADS23*-overexpressing lines. **(A)** Schematic diagram indicating the T-DNA insertion sites in genomic region in *osmads23-1* mutant (M1). Black boxes represent exons; lines between black boxes are introns. The arrow indicates the transcription orientation. **(B)** Quantitative PCR analysis of different regions of *OsMADS23* in *osmads23-1*. **(C)** and **(D)** Phenotypes of wild type (Z11) and *osmads23-1* for 10 and 80 days, respectively. **(E)** Internode morphology in images in (D). **(F)** and **(G)** Quantification of shoot length and internode length in wild type and *osmads23-1*. **(H)** Quantitative PCR analysis of *OsMADS23* in *OsMADS23*-overexpressing lines (OE13 and OE14). **(I)** and **(J)** Phenotypes of wild type (Nip) and *OsMADS23*-overexpressing lines for 10 and 80 days, respectively. **(L)** Internode morphology in images in (J). **(K)** and **(M)** Quantification of shoot length and internode length of Nip and *OsMADS23*-overexpressing lines. The significant difference between *osmads23-1* or *OsMADS23*-overexpressing lines and their corresponding wild type was determined by Student’s *t* test. *p < 0.05, **p < 0.01 or ***p < 0.001. All data displayed as a mean ± SD. M1, *osmads23-1* mutant. In (G) and (M), two-way ANOVA was performed, followed by Bonferroni’s post-hoc test. Different letters indicate significant differences (*p* < 0.05). Three independent experiments were performed (*n* = 30 plants per genotype in each independent experiment).

*OsMADS23* has been reported to be preferentially expressed in the root cylinder in rice previously [38]. In our study, two T-DNA insertion mutant alleles of *OsMADS23*, M1 (*osmads23-1*) and M2 (*osmads23-2*), were obtained, and they have different DNA insertion sites in the third intron of *OsMADS23* (S1A Fig). We found that both M1 (-/-) and M2 (+/-) showed reduced growth indicated by plant height, compared to their corresponding wild type Zhonghua 11 (Z11) (Figs 1A–1G and S1B–S1D). No homozygous seeds of M2 were obtained, possibly because of the DNA deletion in M2 (S1E Fig). Then constitutive expression of *OsMADS23* was performed in rice (Nip) and transgenic rice plants were obtained. Surprisingly, *OsMADS23*-overexpressing lines (OE1~OE20) also exhibited repressed growth (S2 Fig). Two *OsMADS23*-overexpressing lines (OE13 and OE14) were used for further evaluation. The shoot length at seedling stage as well as plant height at maturity in overexpression lines were markedly reduced (Fig 1H–1M), but their agronomic traits such as yield per plant and 1000-grain weight were not changed greatly (S1 Table). These results indicate that *OsMADS23* plays important roles in plant growth and development, and too high or low expression of *OsMADS23* affects plant growth.

### Responses of *osmads23* mutant and *OsMADS23*-overexpressing plants to osmotic stress

*OsMADS23* was greatly induced by PEG, NaCl and mannitol (S3 Fig), which suggests that it is also likely to be crucial for improving plant tolerance to osmotic stress. We therefore investigated the responses of *osmads23* mutant and *OsMADS23*-overexpressing lines to NaCl or PEG in medium, which mimics the salt or drought stress. In control medium for 7 days, overexpression lines grew more slowly than their corresponding wild-type plants (Nip); however, in the medium supplemented with NaCl or PEG, the performance of overexpression lines was remarkably better than that of wild type (S4A–S4C Fig), suggesting that the overexpression lines were less severely affected by osmotic stress than wild type. Expectedly, *osmads23* mutant was substantially more sensitive to NaCl and PEG than its corresponding wild type (Z11) (S4D and S4E Fig), indicating that disruption of *OsMADS23* caused hypersensitivity to osmotic stress in plants. After exposed to NaCl or PEG for 14 days, the difference between overexpression lines and wild type is much more apparent. The shoot growth in wild type was significantly inhibited under osmotic stress conditions, compared with that in untreated plants; however, the repression of shoot length in overexpression lines by NaCl or PEG is not severe (S4F and S4G Fig). The total chlorophyll content, which reflects the rate of chlorosis in seedlings under osmotic stress conditions, was reduced slightly in *OsMADS23*-overexpressing lines, but drastically in wild type, compared with that in their corresponding untreated plants (S4H Fig). These results suggest that OsMADS23 may play an important role in abiotic stress tolerance in plants.

### Overexpression of *OsMADS23* enhances, but disruption of this gene reduces drought tolerance in rice

**Fig 2:**
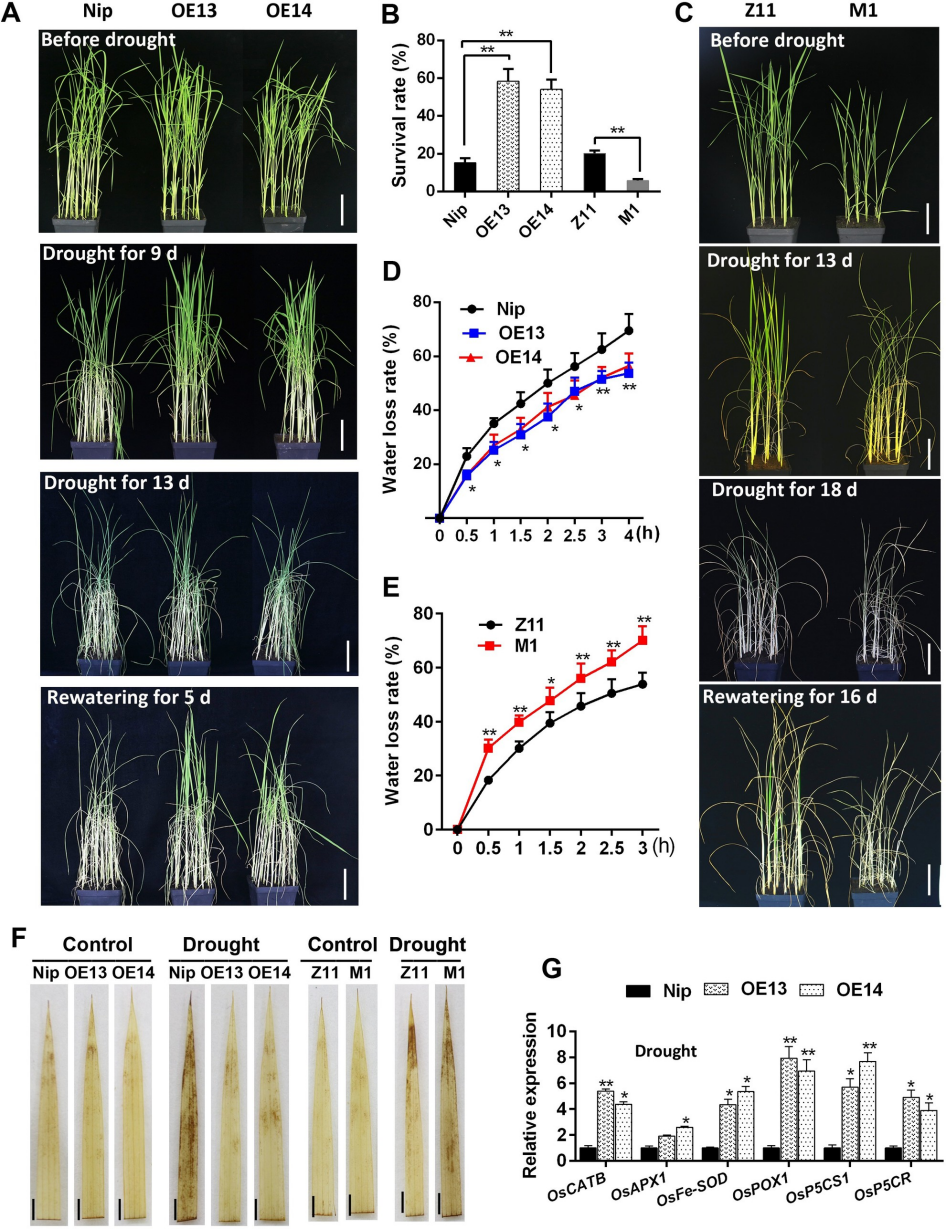
Performance of *osmads23* mutant and *OsMADS23*-overexpressing lines under drought stress. **(A)** Images showing the phenotypes of wild type (Nip) and *OsMADS23*-overexpressing plants (OE13 and OE14) under drought stress. Scale bars, 5 cm. **(B)** The survival rates of overexpression plants or *osmads23-1* mutant (M1) and their corresponding wild type after drought and then rewatering. Error bars indicate SD with biological triplicates (*n* = 3, each replicate containing 48 plants). **(C)** Images showing the phenotypes of wild type (Z11) and *osmads23-1* under drought stress. Scale bars, 5 cm. In (A) and (C), 35-day-old plants were subjected to drought stress and then resumed growth. **(D)** and **(E)** Water loss rates of detached leaves from 70-day-old plants. Error bars indicate SD with biological triplicates (*n* = 3, each replicate containing 5 plants). **(F)** DAB staining for the leaves from rice plants exposed to drought stress for 5 days to indicate H_2_O_2_ levels. Scale bars, 1.5 cm. **(G)** Expression of ROS-scavenging and proline-biosynthetic genes in plants exposed to drought stress for 3 days. Error bars indicate SD with biological triplicates (*n* = 3, each replicate containing 3 plants). The significant difference between *OsMADS23*-overexpressing lines or *osmads23-1* and their corresponding wild-type plants was determined by Student’s *t* test. *p < 0.05, **p < 0.01. All data displayed as a mean ± SD. Three independent experiments were performed.

To further investigate the physiological roles of OsMADS23 in plants, we evaluated the performance of *OsMADS23*-overexpressing lines under drought stress in soil. We found that, after withdrawing water for 9 days, the signs of stress were more severe in wild type (Nip), which exhibited serious chlorosis and wilting of the leaves, whereas the wilting of the *OsMADS23*-overexpressing leaves was delayed (Fig 2A). After a 13-day withdrawing water followed by a 5-day recovery period, over 55% of overexpression lines survived, compared with an about 17% survival rate in wild type (Fig 2B). However, knockout of *OsMADS23* reduced the drought tolerance of plants, and the survival rate of wild type (Z11) (about 20%) was much higher than that of the *osmads23* mutant (about 5%) under the same water stress condition (Fig 2B and 2C). The water loss rate assay also confirmed the result that overexpression lines were more resistant but *osmads23* mutant was more sensitive to drought stress (Fig 2D and 2E). ROS are generally considered to be biomarkers of extensive oxidative stress (Zhang et al. 2015b). Here, the leaf phenotypes differed after DAB staining. Less H_2_O_2_ accumulated in the overexpression lines but more in *osmads23* mutant than that in their corresponding wild type after drought stress (Figs 2F and S5A and S5B). Expectedly, O_2_^-^ levels were similar to H_2_O_2_ (S5C and S5D Fig). Meanwhile, the transcription of genes for ROS-scavenging enzymes was drastically enhanced in overexpression lines under drought stress, compared to wild type (Figs 2G and S5E), which was confirmed by the measurement of antioxidant enzyme activities such as superoxide dismutase (SOD) and catalase (CAT) (S5F and S5G Fig). It is widely accepted that accumulation of osmoprotective solute such as proline is beneficial to regulate the cell osmotic potential under osmotic stress [39]. Here, *OsMADS23*-overexpressing lines had increased expression of *OsP5CS1* and *OsP5CR*, and accumulated more proline than wild type under drought stress (Figs 2G and S5H). MDA is shown as an important indicator of membrane injury and lipid peroxidation caused by environmental stress. After a 5-day withdrawing water, MDA content was significantly increased in wild type, whereas affected less severely in overexpression lines in comparison with that in untreated plants (S5I Fig). These results indicate that OsMASD23 has a positive role in improving plant drought tolerance.

### *OsMADS23* confers rice with the salinity tolerance

**Fig 3:**
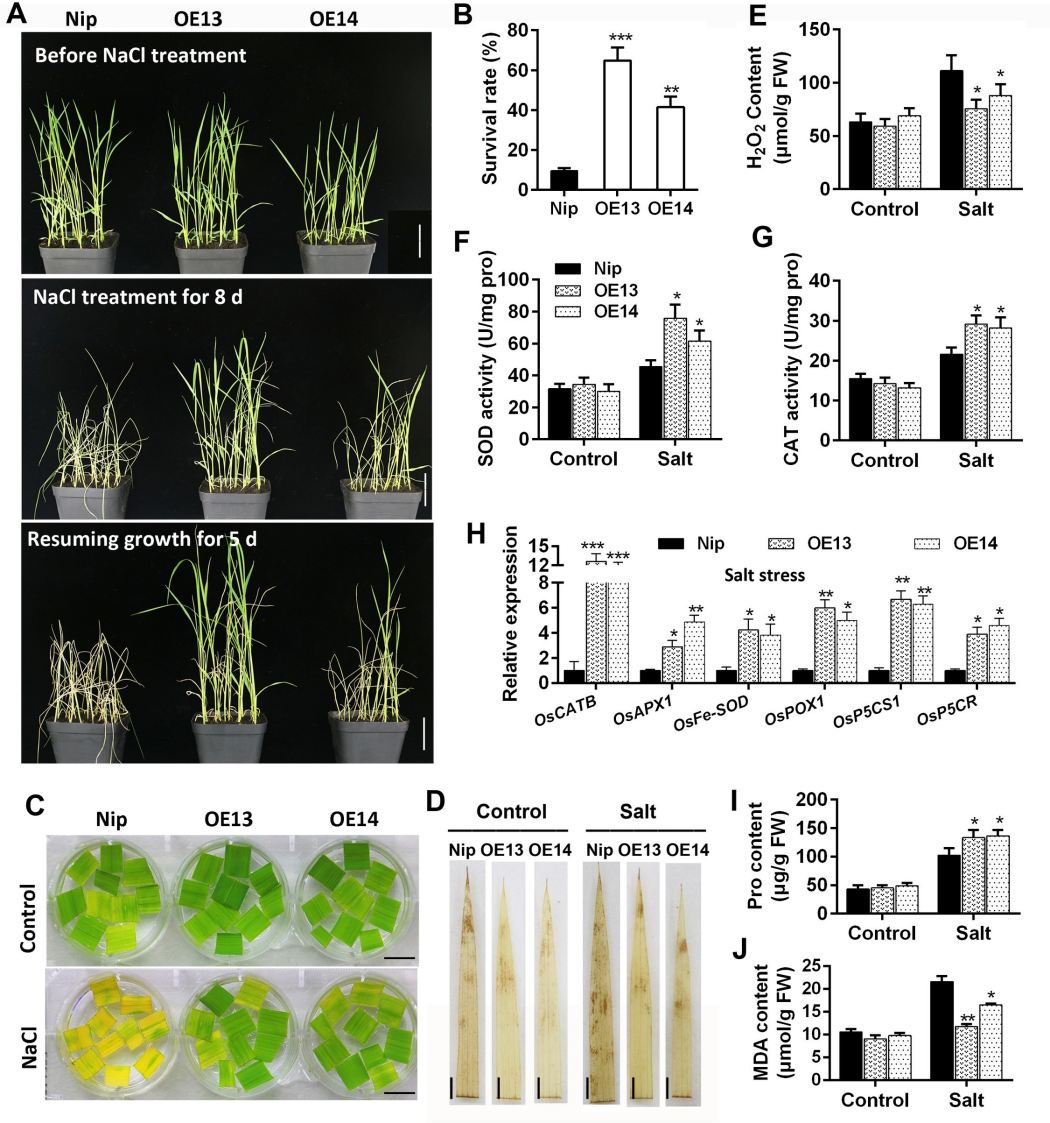
Phenotypes of *OsMADS23*-overexpressing lines under salt stress. **(A)** Images showing the phenotypes of wild type (Nip) and *OsMADS23*-overexpressing lines (OE13 and OE14) under salt stress. Twenty-eight-day-old plants were subjected to 300 mM salt stress and then resumed growth. Scale bars, 5 cm. **(B)** The survival rates of wild type and overexpression lines after 8 days of salt stress and then 5 days of resuming growth. Error bars indicate SD with biological triplicates (*n* = 3, each replicate containing 48 plants). **(C)** The leaves detached from 60-day-old plants were exposed to 200 mM NaCl for 3 days to indicate the salt stress tolerance. Scale bars, 2 cm. **(D)** DAB staining for the leaves of plants exposed to salt stress for 5 days to indicate H_2_O_2_ levels. Scale bars, 1.5 cm. **(E)** Quantification of H_2_O_2_ content in the leaves from plants exposed to salt stress for 5 days. **(F)** and **(G)** Activities of SOD and CAT in plants exposed to salt stress for 5 days, respectively. **(H)** Expression of ROS-scavenging genes in plants exposed to salt stress for 3 days. **(I) and (J)** Content of proline and MDA in plants exposed to salt stress for 5 days, respectively. In (E) to (J), error bars indicate SD with biological triplicates (*n* = 3, each replicate containing 3 plants). *p < 0.05, **p < 0.01 or ***p < 0.001 (Student’s *t* test). All data are means ± SD. Three independent experiments were performed.

In many cases, plants with improved drought tolerance can also resist salt stress [40,41]. In our salt tolerance tests, wild type (Nip) exhibited earlier and more severe wilting symptoms than *OsMADS23*-overexpressing lines, and the latter showed an obvious salt resistance phenotype (Fig 3A). Compared with an about 11% survival rate in wild type, about 50% of overexpression plants survived after a 8-day NaCl treatment followed by a 5-day recovery period (Fig 3B). In parallel to the salt-sensitive phenotype of seedlings, the detached leaves of wild type were observed to bleach more quickly than that of overexpression lines under salt stress (Fig 3C). Expectedly, much less ROS accumulated in overexpression lines after NaCl treatment (Fig 3D and 3E). Moreover, under salt stress, the activities of antioxidant enzymes and transcription of ROS-scavenging genes were significantly enhanced in overexpression plants, compared to that in wild type (Fig 3F–3H). In accordance with the enhanced salt tolerance, proline content was drastically higher but MDA levels were markedly lower in the overexpression lines than that in wild type (Fig 3I and 3J). Together, these data indicate that OsMADS23 is also an essential positive regulator in salt tolerance, and OsMADS23 can enhance the ability of adaption to osmotic stress in plants.

### Overexpression of *OsMADS23* reduces the sensitivity to oxidative stress in plants

**Fig 4:**
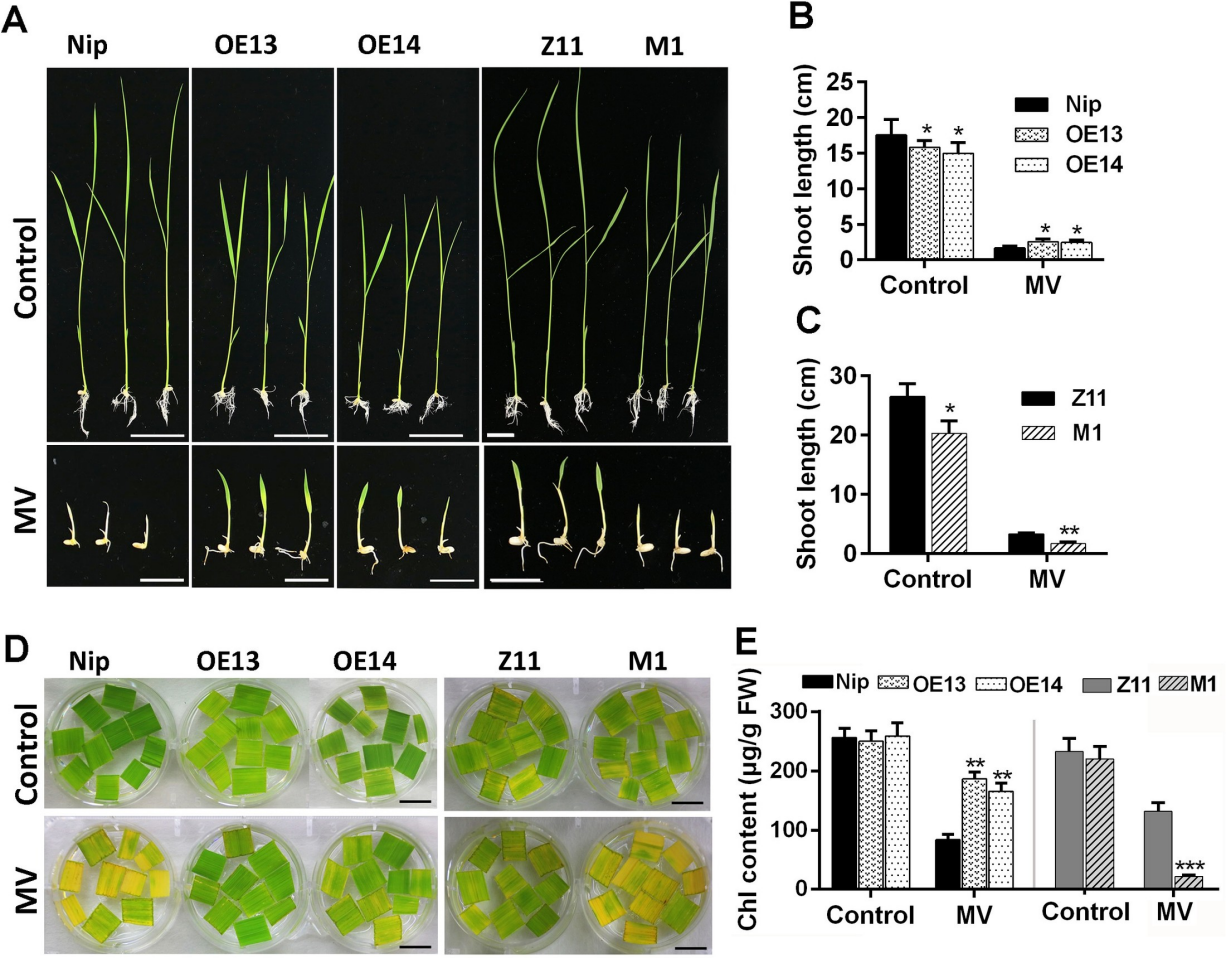
Overexpression of *OsMADS23* promoted plant adaption to oxidative stress. **(A)** Performance of *OsMADS23*-overexpressing plants (OE13 and OE14) or *osmads23-1* mutant (M1) in half-strength medium supplemented with 2 μM MV for 7 days. Two-day-old seedlings were grown in medium with or without MV. Scale bars, 2 cm. **(B)** and **(C)** Shoot length of *OsMADS23*-overexpressing plants and *osmads23-1* mutant in the medium with or without MV for 7 days, respectively. Error bars indicate SD with biological triplicates (*n* = 3, each replicate containing 20 plants). **(D)** The detached leaves from 60-day-old plants were exposed to 5 μM MV for 3 days to indicate the oxidative tolerance. Scale bars, 1.5 cm. **(E)** The chlorophyll content of 3-day-old plants growing in the medium with or without MV for 7 days, respectively. Error bars indicate SD with biological triplicates (*n* = 3, each replicate containing 3 plants). MV, methyl viologen. *p < 0.05, **p < 0.01 or ***p < 0.001 (Student’s *t* test). All data are means ± SD. Three independent experiments were performed.

The increased drought and salt resistance of *OsMADS23*-overexpressing plants (Figs 2 and 3) suggests that they might have the enhanced tolerance to oxidative stress. To further confirm this, the response of *OsMADS23*-overexpressing lines to oxidative stress was investigated by using MV, an oxidative stress inducer in plants. Two-day-old seedlings were grown on half-strength MS medium supplemented with 2 mM MV. In the medium without MV, overexpression plants grow more slowly than wild type; however, after 7 days of growth in MV, the growth impairment in wild type (Nip) was much more severe than that of overexpression lines, indicated by the shoot length (Fig 4A and 4B). On the contrary, *osmads23* mutant was much more hypersensitive to oxidation stress than its corresponding wild type (Z11) (Fig 4A and 4C). Oxidation can cause degradation of chlorophyll and etiolating phenotypes [42]. Here, the detached leaves of *OsMADS23*-overexpressing plants exhibited much less sensitivity to oxidative stress, whereas *osmads23* mutant had a quicker bleaching rate than its corresponding wild type (Fig 4D). After MV treatment, wild type (Nip) showed a severe reduction of chlorophyll (only 30% of chlorophyll of untreated plants retained), whereas the chlorophyll content of overexpression plants just decreased slightly (about 70% of untreated plants retained) (Fig 4E). By contrast, *osmads23* mutant had more significant chlorophyll reduction than its wild type (Z11) (Fig 4E). These results further confirmed that OsMADS23 positively regulated the oxidation tolerance in plants, and its overexpression can attenuate oxidative damage under oxidative stress, suggesting that *OsMADS23* is a promising candidate gene for improving the oxidation tolerance in plants.

### *OsMADS23* mediates ABA sensitivity and is involved in ABA-induced stomatal closure in plants

**Fig 5:**
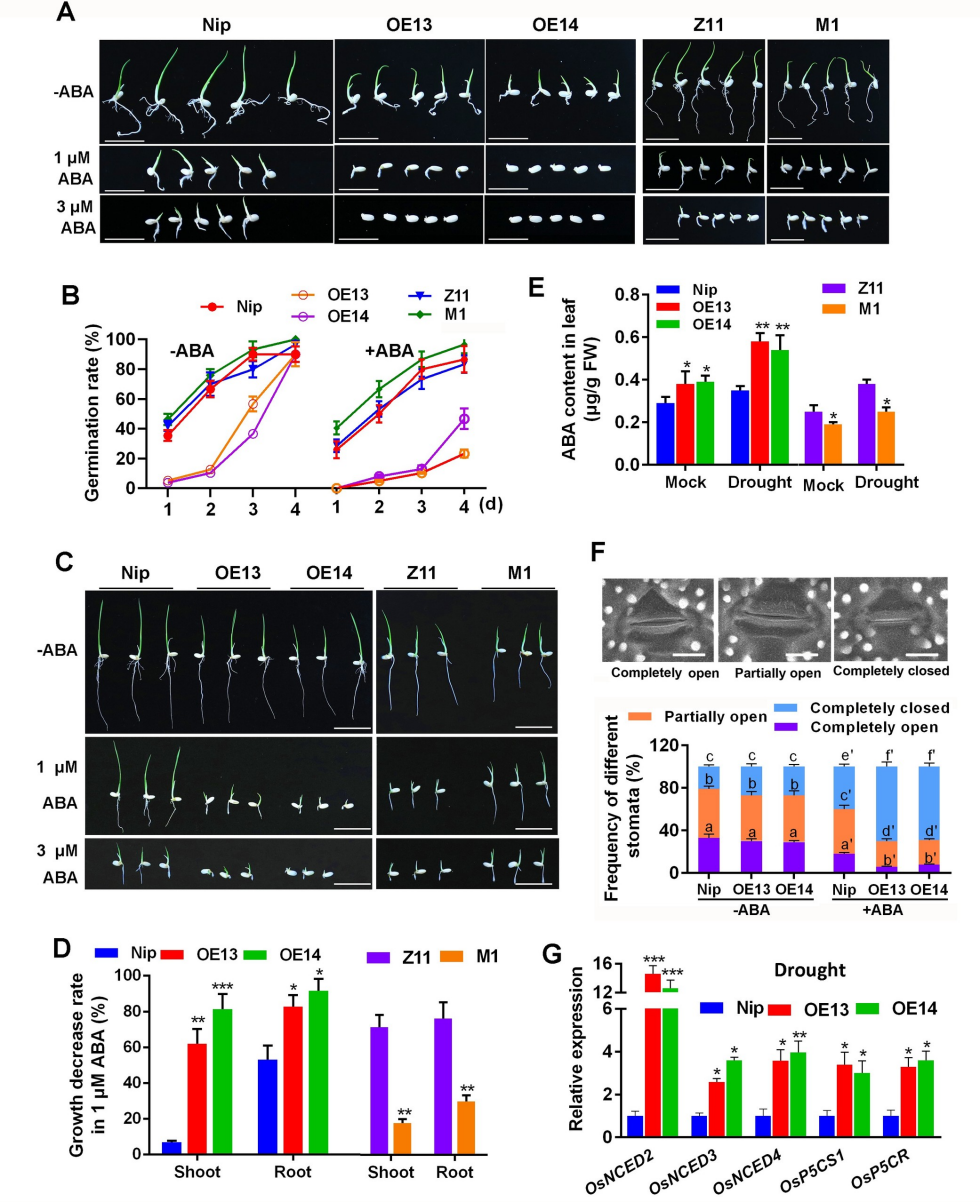
OsMADS23 mediates ABA sensitivity and ABA-induced stomatal movement in rice. **(A)** Seed germination of *OsMADS23*-overexpressing plants (OE13 and OE14) or *osmads23-1* mutant (M1) compared to their corresponding wild type (Nip or Z11) on half-strength MS medium without or with ABA for 4 days, respectively. Scale bars, 2 cm. **(B)** Seed germination rates scored from day 1 to day 4 after stratification on medium supplemented without or with 1 μM ABA, respectively. Error bars indicate SD with biological triplicates (*n* = 3, each replicate containing 50 seeds). **(C)** Plant growth in half-strength MS medium without or with ABA for 4 days, respectively. Two-day-old seedlings were transferred on medium with or without ABA. Scale bars, 2 cm. **(D)** Decrease rate of shoot and root length in 1 μM ABA compared to mock on day 4. Error bars indicate SD with biological triplicates (*n* = 3, each replicate containing 20 plants). **(E)** ABA content in *OsMADS23*-overexpressing lines and *osmads23* mutant, and their corresponding wild type after exposed to drought stress. Two-week-old plants were subjected to drought stress for 3 days, and leaves were collected for measurement of ABA content. Error bars indicate SD with biological triplicates (*n* = 3, each replicate containing 5 plants). **(F)** Percentages of completely open, partially open, and completely closed stomata in wild type (Nip) and *OsMADS23*-overexpressing plants under normal and ABA treatment conditions. Results represent means ± SD (*n* = 300) from 10 plants per genotype. Two-way ANOVA was performed, followed by Bonferroni’s post-hoc test. Scale bars, 10 μm. **(G)** Relative transcription levels of key genes involved in ABA*-*dependent stress response pathway in 2-week-old plants under drought conditions for 3 days. Error bars indicate SD with biological triplicates (*n* = 3, each replicate containing 3 plants). *p < 0.05, **p < 0.01 or ***p < 0.001 (Student’s *t* test). Three independent experiments were performed.

ABA has been widely considered as a stress hormone, and plant drought and salt responses are closely related to ABA sensitivity [7,30,43]. To further investigate whether *OsMADS23* is involved in ABA responses, we investigated the seed germination as well as shoot and primary root (PR) elongation in different genotypes of *OsMADS23* in response to exogenous ABA. Compared to control, both the seed germination and plant growth in *OsMADS23*-overexpressing lines were repressed more severely, whereas was much less severe in *osmads23* mutant than their corresponding wild type (Figs 5A–5D and S6A–S6F). The results indicate that overexpression of *OsMADS23* increases the sensitivity of plants to exogenous ABA, and suggest its potential role in ABA signaling. Meanwhile, the endogenous ABA accumulation in plants in response to drought stress was evaluated. As shown in Fig 5E, after drought for 3 days, the ABA levels were much higher in overexpression plants, but lower in *osmads23* mutant than their corresponding wild type.

It is well recognized that ABA promotes stomatal closure to avoid water loss under drought or salt stress. To explore possible cellular processes affected by *OsMADS23* in improving plant osmotic stress tolerance, we compared the ABA-induced stomatal movement in *OsMADS23*-overexpressing lines with that in wild type (Nip). Under daylight conditions, stomata in rice leaves can be classified into three typical status categories: completely open, partially open, and completely closed [44] (Fig 5F). In the absence of ABA, there was little difference in the proportions of the three categories of stomata between overexpression lines and wild type (Fig 5F). However, after ABA treatment, the proportions of the completely and partially open stomata in overexpression lines were significantly lower than that in wild type, while the proportion of the completely closed stomata was markedly higher (Fig 5F). Hence, we concluded that *OsMADS23* was involved in ABA-induced stomatal closure. Elevated endogenous ABA accumulation as well as ABA-induced stomatal closure suggests that the expression of ABA-responsive genes might be altered in overexpression plants. To verify this supposition, expression of stress-inducible marker genes that function in the ABA-dependent pathway was analyzed. As shown in Figs 5G and S6G, compared to mock, the expression of ABA-biosynthetic genes such as *OsNCED2*, *OsNCED3*, *OsNCED4* and ABA-inducible genes *OsP5CR* and *OsP5CS1* in overexpression lines was much higher than that in wild type under drought conditions. Together, these results clearly show that OsMADS23 regulates the drought and salt tolerance in plants, at least partially, through the ABA-dependent pathway.

### OsMADS23 directly activates the transcription of *OsNCEDs* and *OsP5CR* through binding the CArG-box motifs

**Fig 6:**
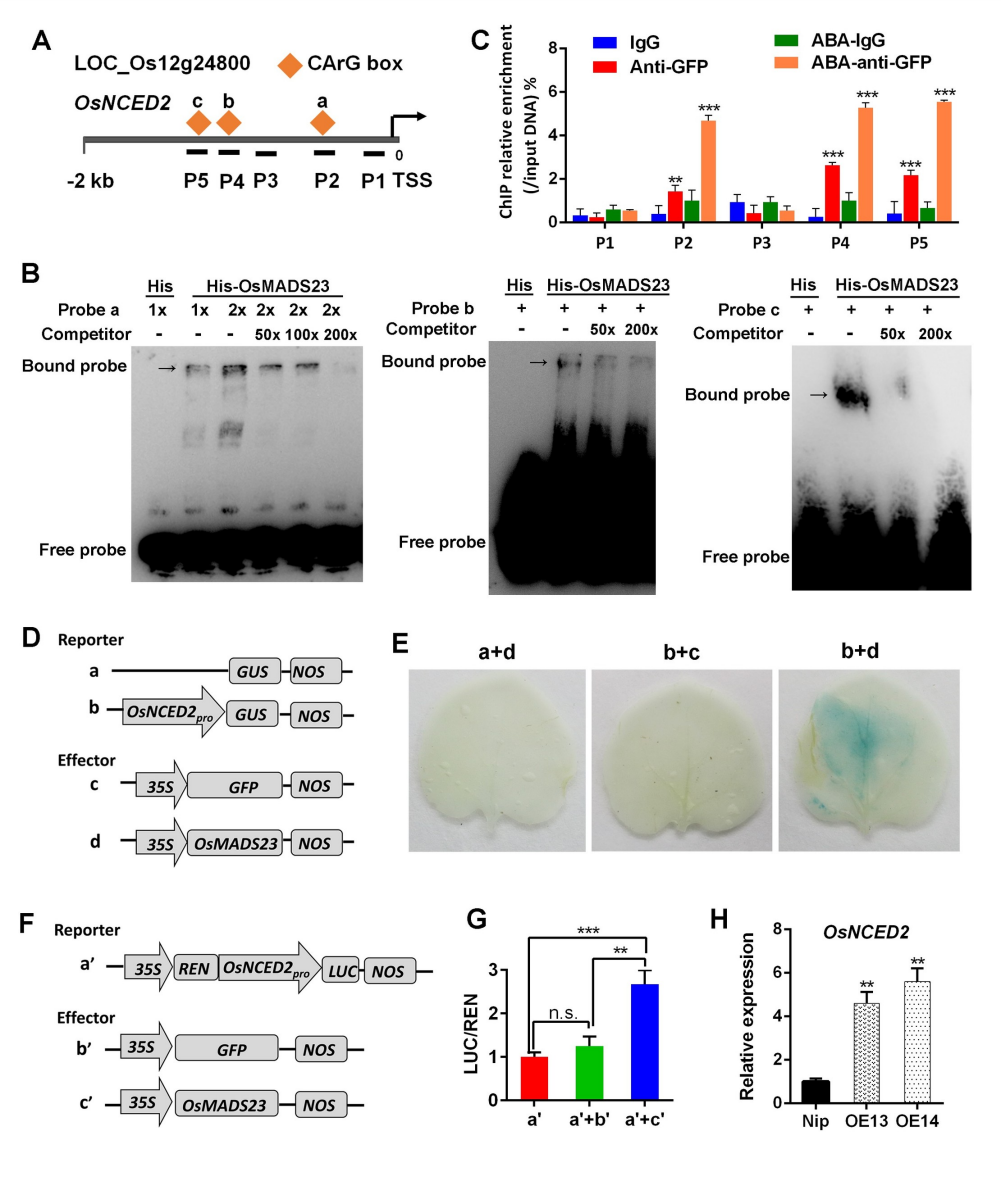
OsMADS23 is a transcriptional activator of *OsNCED2*. **(A)** Schematic diagram of *OsNCED2* promoter region showing the positions of CArG-box motifs and P1-P5 fragments amplified by ChIP-qPCR. **(B)** Electrophoretic mobility shift assays (EMSA) indicating OsMADS23 binds the CArG-box motifs in *OsNCED2* promoter specifically. Probe 1 (-1266 to -1226 bp), probe 2 (-1051 to -1010 bp) and probe 3 (-687 to -645 bp). **(C)** Chromatin immunoprecipitation-quantitative PCR (ChIP-qPCR) assay showed that ABA enhances OsMADS23 binding to the promoter regions of *OsNCED2*. P1-P5 represents the regions shown in (A) detected by ChIP-qPCR. The enrichment values were normalized to input. Immunoglobulin G (IgG) immunoprecipitated DNA was used as a control. Error bars indicate SD with biological triplicates. Ten-day-old rice plants overexpressing OsMADS23-GFP were treated by ABA for 16 h and used for ChIP analysis. Error bars indicate SD with biological triplicates. **(D)** Schematic diagrams of the effector and reporter used for transient transactivation assays in the leaves of *Nicotiana* *benthamiana*. The fragment from -1362 to -574 bp in the promoter of *OsNCED2* was used for constructing reporter. **(E)** Transactivation activity was detected by GUS staining in *N*. *benthamiana* leaves. **(F)** Schematic diagrams of the effector and reporter used for transient transactivation assays in rice protoplasts. REN, Renilla luciferase; LUC, firefly luciferase. The fragment from -1362 to -574 bp in the promoter of *OsNCED2* was used for construction reporter. **(G)** Transactivation activity reflected by LUC activity of LUC/REN ratio. Data represent the means of three independent experiments. **(H)** Relative transcription levels of *OsNCED2* in wild type (Nip) and *OsMADS23*-overexpressing plants. *p < 0.05, **p < 0.01 or ***p < 0.001 (Student’s *t* test). Three independent experiments were performed.

*OsMADS23*-overexpressing plants accumulated much higher levels of ABA than wild type in response to drought stress (Fig 5E), and the transcripts of ABA biosynthetic genes, especially *OsNCED2*, were also shown to be more abundant in overexpression plants than that in wild type under drought stress (Fig 5G), suggesting that *OsNCEDs* might be the targets of OsMADS23. Three CArG-box motifs were found in the *OsNCED2* promoter region (Fig 6A). First, we examined whether OsMADS23 specifically binds to these CArG-box motifs *in vitro* by using electrophoretic mobility shift assay (EMSA). As shown in Fig 6B, when His-OsMADS23 was incubated with these motifs (probe a, b and c), respectively, there was obvious gel retardation in the three labeled probes, demonstrating that OsMADS23 can bind to the *OsNCED2* promoter region. The intensity of binding signal was clearly reduced by adding excessive corresponding unlabeled probe (competitor) in the reaction, indicating that OsMADS23 binding to the promoter region of *OsNCED2* was specific (Fig 6B). To further determine whether OsMADS23 binds to the *OsNCED2* promoter *in vivo*, we performed chromatin immunoprecipitation quantitative PCR (ChIP-qPCR) assays. GFP-Trap beads binding with OsMADS23-GFP were used for coimmunoprecipitation of the associated DNA fragments from the leaves of *OsMADS23-GFP* transgenic plants with or without ABA treatment. Quantitative PCR analysis was performed to quantify the enrichment of specific DNA regions of the *OsNCED2* promoter precipitated by the GFP antibody. In support of EMSA results, significant enrichment was observed in the DNA regions of the *OsNCED2* promoter containing CArG-boxes, while no enrichment was found in other regions (Fig 6A and 6C). Notably, these enrichment was increased greatly after plants were treated by ABA (Fig 6A and 6C), indicating the positive effect of ABA treatment on the transcriptional activity of OsMADS23. To further verify the regulation of *OsNCED2* by OsMADS23, we performed transient transactivation assay in *Nicotiana benthamiana* leaves. GUS staining showed that *GUS* can be expressed only when *35S*::*OsMADS23* and *OsNCED2*_*Pro*_::*GUS* were coinfiltrated (Fig 6D and 6E). Expectedly, this result was also confirmed using LUC/REN assay in rice protoplasts. Transient coexpression of OsMADS23 with *OsNCED2*_*Pro*_::*LUC* in protoplasts significantly increased the LUC/REN ratio (Fig 6F and 6G). As a fact, we also found the increased transcription of *OsNCED2* in *OsMADS23*-overexpressing lines (Fig 6H). Thus, our data indicate that OsMADS23 is an upstream transcriptional activator of *OsNCED2* and regulates its expression *in vivo*.

**Fig 7:**
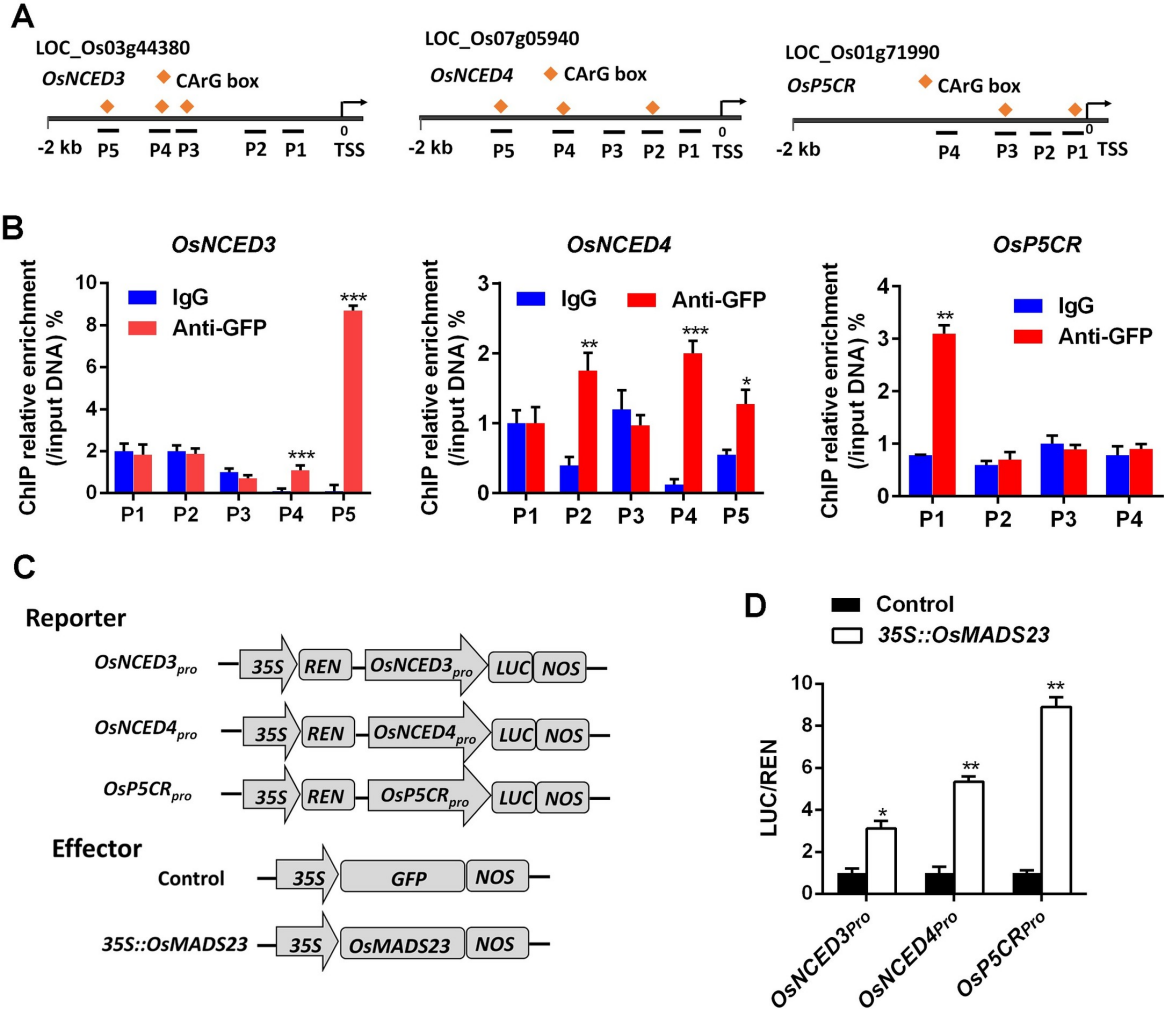
OsMADS23 binds to the promoters of *OsNCED3*, *OsNCED4* and *OsP5CR*, and activates their expression *in vivo*. **(A)** Schematic diagrams of *OsNCED3*, *OsNCED4* and *OsP5CR* showing the positions of CArG-box motifs and fragments amplified by ChIP-qPCR, respectively. **(B)** ChIP-qPCR analysis of the gene fragments of *OsNCED3*, *OsNCED4* and *OsP5CR* enriched by OsMADS23 in rice plants, respectively. The enrichment values were normalized to input. Immunoglobulin G (IgG) immunoprecipitated DNA was used as a control. Ten-day-old rice plants overexpressing OsMADS23-GFP were used for ChIP analysis. Error bars indicate SD with biological triplicates. **(C)** Schematic diagrams of the effector and reporter used for transient transactivation assays in rice protoplasts. REN, Renilla luciferase; LUC, firefly luciferase. The fragments in the promoter regions of *OsNCED3* (-1964 to -1740 bp), *OsNCED4*, (-1349 to -1100 bp) and *OsP5CR* (-686 to -781 bp) were used for construction of reporters. **(D)** Transactivation activity reflected by LUC activity of LUC/REN ratio in rice protoplasts. Error bars indicate SD with biological triplicates. *p < 0.05, **p < 0.01 or ***p < 0.001 (Student’s *t* test).

Recent study has shown that overexpression of *OsNCED3* or *OsNCED4* in plants can enhance water stress tolerance by increasing ABA levels [25,45]. In our study, proline accumulated drastically in response to drought and salt stress in *OsMADS23*-overexpressing lines (Figs 3I and S5H), hinting OsMADS23 might activate the expression of proline synthetic genes such as *OsP5CR* or *OsP5CS1* to modulate stress tolerance. To test whether OsMADS23 regulates the expression of ABA or proline synthetic genes *in vivo*, we performed ChIP-qPCR and transient transactivation assays, and the results showed that OsMADS23 could directly activate the transcription of *OsNCED3*, *OsNCED4* and *OsP5CR*, respectively, by specifically recognizing the CArG-box motifs in their promoter regions (Fig 7). These results demonstrate that OsMADS23 increases the osmotic stress tolerance by activating the expression of the key genes functioning in ABA and proline synthesis.

### *osnced2* mutants exhibit reduced tolerance to drought and oxidation stress

**Fig 8:**
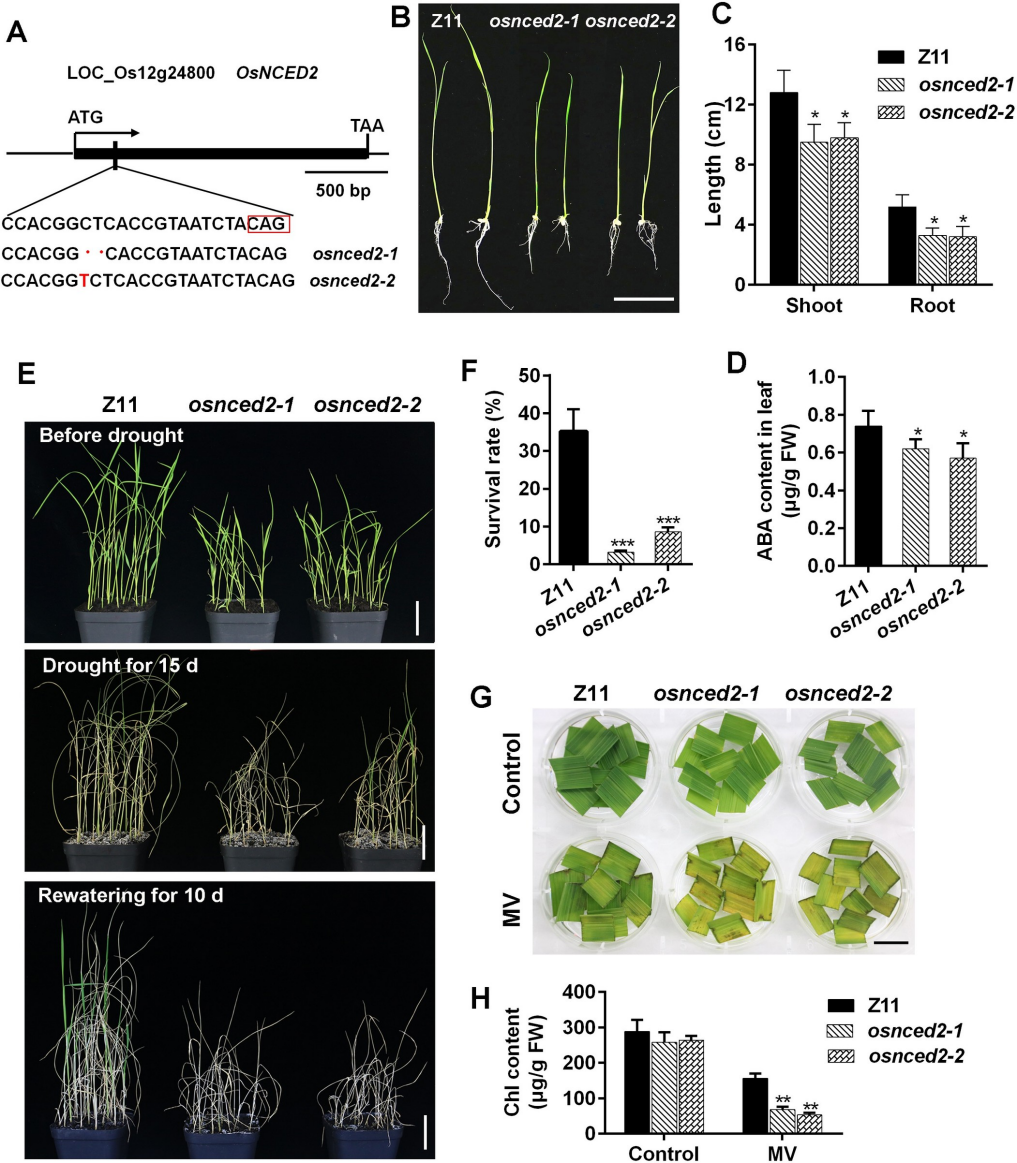
*osnced2* mutants were more sensitive to drought and oxidation stress than wild type. **(A)** Schematic diagram of CRISPR-Cas9-mediated target mutagenesis of *OsNCED2*. **(B)** Phenotypes of *osnced2* mutants and wild type (Z11) in half-strength medium for 7 days. Scale bars, 4 cm. **(C)** Shoot and root length of wild type and *osnced2* mutants in half-strength medium for 7 days. Error bars indicate SD with biological triplicates (*n* = 3, each replicate containing 20 plants). **(D)** ABA content in the leaves of wild type (Z11) and *osnced2* mutants growing for 20 days. Error bars indicate SD with biological triplicates (*n* = 3, each replicate containing 3 plants). **(E)** Images showing the phenotypes of wild type and *osnced2* mutants under drought stress. Twenty-day-old plants were subjected to drought stress and then rewatering. Scale bars, 5 cm. **(F)** The survival rates of wild type and *osnced2* mutants after drought stress and rewatering. Error bars indicate SD with biological triplicates (*n* = 3, each replicate containing 48 plants). **(G)** The detached leaves from 70-day-old plants were exposed to 5 μM MV for 3 days to indicate the oxidative tolerance. Scale bars, 2 cm. **(H)** The chlorophyll content of wild type and *osnced2* mutants in MV for 7 days. Error bars indicate SD with biological triplicates (*n* = 3, each replicate containing 3 plants). MV, methyl viologen. *p < 0.05, **p < 0.01 (Student’s *t* test). Three independent experiments were performed.

Having elucidated OsMADS23 regulates the osmotic stress tolerance by regulating ABA and proline biosynthesis via binding to the promoter regions of *OsNCED2*, *OsNCED3*, *OsNCED4*, and *OsP5CR*, we are interested in their potential contribution to osmotic stress resistance. *OsNCED3* and *OsNCED4* have been shown to promote ABA biosynthesis and abiotic stress tolerance [25,45]. *OsP5CR* is found to play crucial roles in salt tolerance [37,46]. The roles of *OsNCED2* in drought stress tolerance in plants still remain elusive. Here we explored the contribution of *OsNCED2* to water stress tolerance by using two independent homozygous mutants (*osnced2-1* and *osnced2-2*, Z11 background) produced by CRISPR/Cas9 system. *osnced2-1* has a two-nucleotide deletion, and *osnced2-2* has a one-nucleotide insertion in the position of 87 bp after ATG, respectively, and these lead to frameshift mutations that promote early termination of protein translation (Figs 8A and S7A). *OsNCED2*, which has only one exon of 1710 bp in length, was constitutively expressed in various tissues in rice plants (S7B Fig). Knockout of *OsNCED2* significantly impaired plant growth, indicated by the reduced shoot and root length (Fig 8B and 8C). Given NCED as the key rate-limiting enzyme in ABA biosynthesis, therefore, we first investigated the ABA accumulation in *osnced2* mutants. As shown in Fig 8D, the ABA levels were significantly reduced in *osnced2* mutants compared to that in wild type (Z11). Then, the contribution to the drought stress tolerance made by *OsNCED2* was evaluated. After withdrawing water and then rewatering, approximately 35.2% of the wild type plants survived, but only 3.2% of *osnced2-1* and 8.6% of *osnced2-2* plants recovered, respectively (Fig 8E and 8F). The water stress sensitivity of the *osnced2* mutants was confirmed by the oxidation stress assay. When exposed to MV, the detached leaves of *osnced2* mutants were observed to bleach more quickly than that of wild type (Fig 8G). Additionally, the *osnced2* mutants had more severe decrease of chlorophyll than wild type in the presence of MV (Fig 8H). These results indicate that *osnced2* mutants have a reduced capacity for osmotic and oxidant stress tolerance.

### OsMADS23 physically interacts with SAPK9

**Fig 9:**
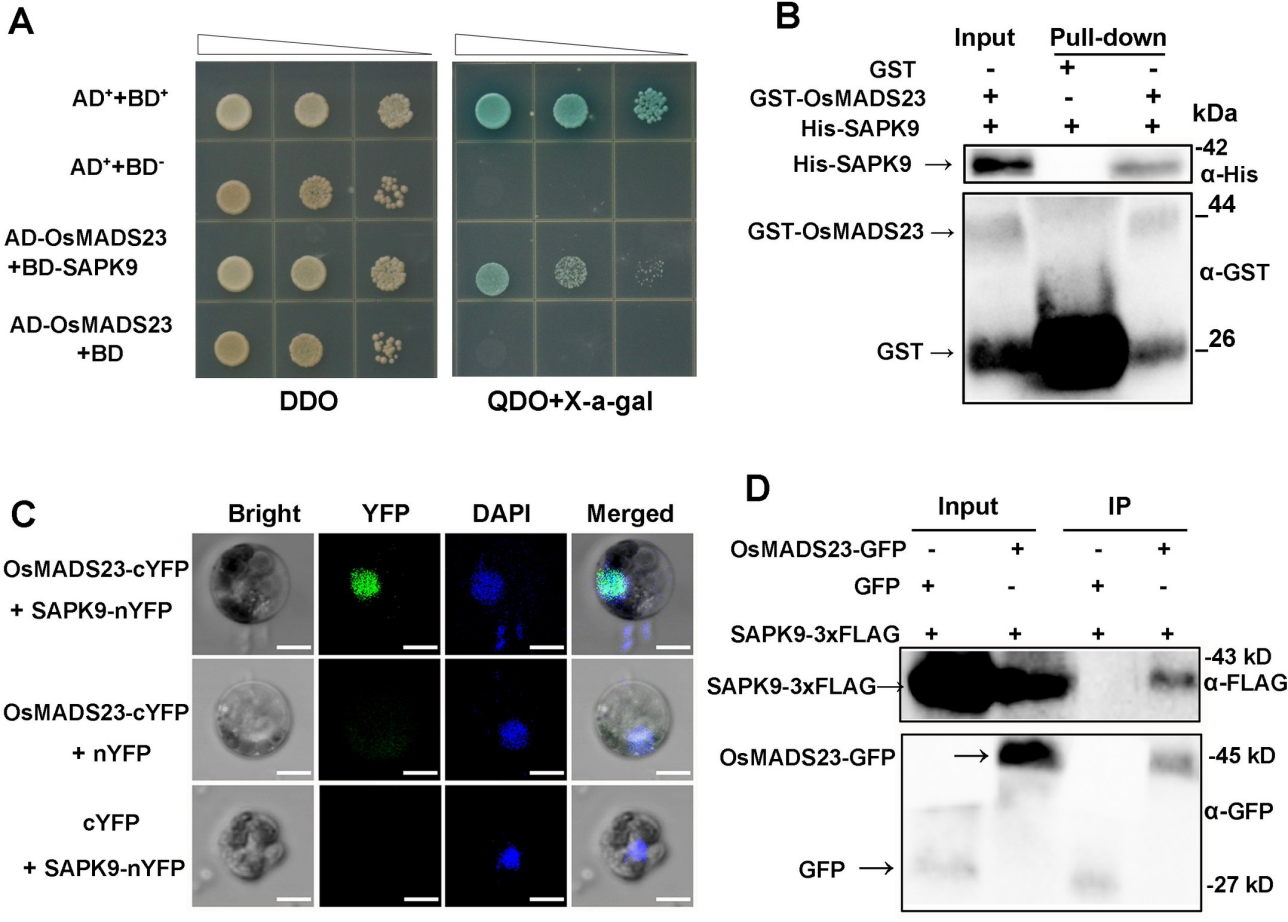
OsMADS23 physically interacts with SAPK9. **(A)** Yeast two-hybrid assays of OsMADS23 and SAPK9. SD, synthetic dropout medium. DDO, SD/-Leu-Trp. QDO, SD/-Ade-His-Leu-Trp. **(B)** OsMADS23 interacts with SAPK9 in the *in vitro* pull-down assay. GST-tagged OsMADS23 (GST-OsMADS23) was used as a bait, and pull-down of His-SAPK9 was detected by anti-His antibody (Proteintech). **(C)** The interaction of OsMADS23 and SAPK9 by bimolecular fluorescence complementation (BiFC) in rice protoplasts. YFP, Yellow fluorescent protein. Scale bars, 5 μm. **(D)** OsMADS23-SAPK9 interaction detected by coimmunoprecipitation (CoIP) assay. Total proteins from *Nicotiana* *benthamiana* leaves coexpressing OsMADS23-GFP with SAPK9-3×FLAG or 3×FLAG were used. Proteins before (input) and after IP were detected by the anti-GFP and anti-FLAG antibodies (Proteintech), respectively. Three independent experiments were performed.

Rice SnRK2s such as SAPK8, SAPK9 and SAPK10 are activated by ABA, and had crucial roles in abiotic stress tolerance [13,47,48], which promotes us to test whether OsMADS23 can physically interact with these SAPKs. OsMADS23 was localized to the nucleus (S8 Fig). Yeast two-hybrid assays showed that SAPK9 interacted with OsMADS23 (Fig 9A), but not SAPK8 or SAPK10 (S9 Fig). GST pull-down assay showed that GST-OsMADS23, but not GST alone, pulled down a significant amount of His-SAPK9 (Fig 9B). The direct interaction between SAPK9 and OsMADS23 was further confirmed by bimolecular fluorescence complementation (BiFC) in rice protoplasts and coimmunoprecipitation (CoIP) assays in *N*. *benthamiana*, respectively. In the BiFC assay, a strong fluorescence signal was observed in the nucleus of rice protoplast coexpressing SAPKP9-nYFP and OsMADS23-cYFP, but no signal was detected when each construct was coexpressed with an empty vector (Fig 9C). In addition to the BiFC assay, the SAPK9-OsMADS23 interaction was corroborated by CoIP assay (Fig 9D). These results demonstrate that OsMADS23 physically interacts with SAPK9 in the nucleus in rice protoplasts.

### SAPK9 phosphorylates OsMADS23 *in vitro* and *in vivo*

**Fig 10:**
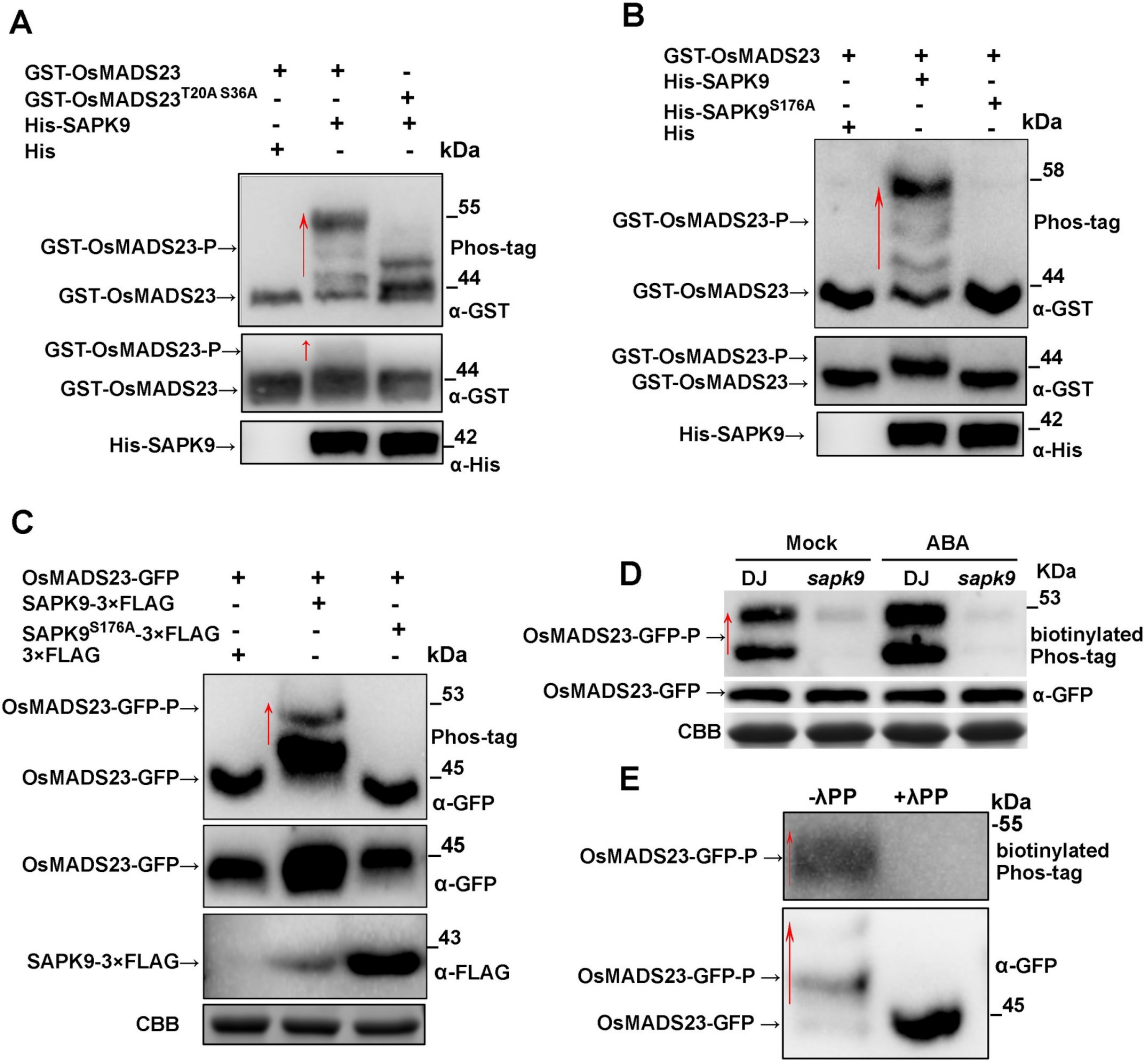
SAPK9 phosphorylates OsMADS23 *in vitro* and *in vivo*. **(A)** Thr-20 and Ser-36 in OsMADS23 are main sites of SAPK9-mediated phosphorylation. Kinase assays *in vitro* were performed by coexpressing His-SAPK9 with the mutated form of GST-OsMADS23 containing both putative phosphorylation sites substituted with Ala in *Escherichia coli* BL21 (DE3). GST-OsMADS23 and phosphorylated GST-OsMADS23 (GST-OsMADS23-P) were purified and detected using a Phos-tag gel by anit-GST (top panel). An equal amount of each recombinant protein was separated on the gel without the Phos-tag as a loading control, detected by anti-GST (middle panel) and anti-His (bottom panel), respectively. **(B)** Serine 176 is the key phosphorylation site of SAPK9 on OsMADS23. Kinase assays were performed by coexpressing GST-OsMADS23 with His-SAPK9 or His-SAPK9^S176A^ in BL21 (DE3). GST-OsMADS23 and GST-OsMADS23-P were purified and detected using a Phos-tag gel by anit-GST (top panel). An equal amount of each recombinant protein was separated on the gel without the Phos-tag as a loading control, detected by anti-GST (middle panel) and anti-His (bottom panel), respectively. **(C)** OsMADS23-GFP was phosphorylated by SAPK9-3×FLAG in *Nicotiana* *benthamiana* leaves. OsMADS23-GFP was transiently coexpressed with empty FLAG protein (3×FLAG), SAPK9-3×FLAG, or SAPK9^S176A^-3×FLAG in 3-week-old *N*. *benthamiana* leaves by *Agrobacterium* infiltration. An equal amount of each total proteins from tobacco leaves was detected using a Phos-tag gel by anit-GFP (top panel). An equal amount of each protein was separated on the gel without the Phos-tag, detected by anti-GFP (middle panel) and anti-FLAG (bottom panel), respectively. Coomassie brilliant blue (CBB) staining indicates similar amounts of proteins were loaded. **(D)** Phosphorylation of OsMADS23-GFP in DJ (wild type) and *sapk9* mutant. OsMADS23-GFP was transiently expressed in the protoplasts of DJ or *sapk9* mutant, and then was immunoprecipitated with anti-GFP and detected with biotinylated Phos-tag (Phosbind biotin BTL-104, APE×BIO). An equal amount of protein extracts were separated on the gel without the Phos-tag and detected by anti-GFP (middle panel). Coomassie brilliant blue (CBB) staining indicates similar amounts of proteins were loaded. To avoid protein degradation, MG132 and a cocktail of proteinase inhibitors were added. **(E)** Immunoprecipitated OsMADS23-GFP protein from OsMADS23-GFP rice plants was treated with or without Lambda protein phosphatase (λ PP). The phosphorylated proteins were detected with biotinylated Phos-tag (Phosbind biotin BTL-104, APE×BIO) (top panel). An equal amount of immunoprecipitated OsMADS23-GFP protein was separated on the gel without the Phos-tag as a loading control, and detected by anti-GFP (bottom panel). Red arrows represent retarded phosphorylated protein. To avoid protein degradation, MG132 and a cocktail of proteinase inhibitors were added. In these west blot experiments, ani-His, anti-GST, anti-GFP and anti-FLAG antibodies (Proteintech) were used. Three independent experiments were performed. Red arrows indicate phosphorylated proteins.

Given the kinase feature of SAPK9, the OsMADS23-SAPK9 interaction urges us to check kinase-substrate relationship between them. First, the kinase assay *in vitro* was performed. As shown in Fig 10A, we detected the phosphorylated GST-OsMADS23 (GST-OsMADS23-P) when coexpressing GST-OsMADS23 with His-SAPK9 in BL21 (DE3), but not His alone, indicating GST-OsMADS23 can be phosphorylated by His-SAPK9 (top panel, middle lane). Two R-X-X-S/T and R-Q-X-S/T conversed motifs recognized by SnRK2s were found in OsMADS23 (S10 Fig). To further confirm the phosphorylation sites recognized by SAPK9 in OsMADS23, the mutated form of OsMADS23 with putative phosphorylation sites substituted with Ala was used to perform kinase assays. We found that His-SAPK9 could phosphorylate GST-OsMADS23^T20A S36A^ (Thr-20 to Ala, Ser-36 to Ala), but its phosphorylation level was significantly reduced (Fig 10A, top panel, right lane), indicating that Thr-20 and Ser-36 in OsMADS23 are main phosphorylation sites recognized by SAPK9. In addition to Thr-20 and Ser-36, there might be other phosphorylation sites recognized by SAPK9 in OsMADS23, because phosphorylated bands still can be detected when GST-OsMADS23^T20A S36A^ was coexpressed with His-SAPK9 (Fig 10A, top panel, right lane). Recent research has shown that SAPK10 exhibits autophosphorylation activity on Ser-177 [49], which corresponds to Ser-176 in SAPK9, based on the sequence similarity analysis. To further confirm OsMADS23 phosphorylation mediated by SAPK9, SAPK9^S176A^ (Ser-176 to Ala) was used to perform kinase assays *in vitro*. We found that His-SAPK9^S176A^ could hardly phosphorylate GST-OsMADS23 (Fig 10B, top panel, right lane), suggesting that Ser-176 is crucial for the kinase activity for SAPK9. To further test whether OsMADS23 is phosphorylated by SAPK9 *in vivo*, OsMADS23-GFP was transiently coexpressed with 3×FLAG, SAPK9-3×FLAG, or SAPK9^S176A^-3×FLAG in *N*. *benthamiana* leaves. As shown in Fig 10C (top panel), phosphorylated OsMADS23-GFP (OsMADS23-GFP-P) was detected only coexpressing OsMADS23-GFP with SAPK9-3×FLAG, demonstrating that OsMADS23-GFP was phosphorylated by SAPK9-3×FLAG, but not by SAPK9APK9^S176A^-3×FLAG *in vivo*. Moreover, we found that the accumulation of OsMADS23-GFP was greatly increased in plants in the presence of SAPK9-3×FLAG (Fig 10C, middle panel), suggesting OsMADS23 phosphorylation mediated by SAPK9 stabilizes the protein. OsMADS23 phosphorylation by SAPK9 was further confirmed in rice plants. Phosphorylated OsMADS23-GFP detected by biotinylated Phos-tag in the loss-of-function mutant *sapk9* accumulated much less than that in wild type (DJ), and ABA treatment increased OsMADS23-GFP phosphorylation in wild type, but not in *sapk9* mutant (Figs 10D and S11). Meanwhile, phosphorylated OsMADS23-GFP in *OsMADS23*-overexpressing rice plants was detected, but no phosphorylated protein was detected after adding Lambda protein phosphatase (λ PP) (Fig 10E, top panel). At the same time, when detected with anti-GFP antibody, phosphorylated OsMADS23-GFP was detected, while only OsMADS23-GFP (dephosphorylated) was detected after adding λ PP (Fig 10E, bottom panel). Taken together, these results illustrate that OsMADS23 is phosphorylated by SAPK9, and ABA increases OsMADS23 phosphorylation in rice plants.

### SAPK9 enhances the stability and transcriptional activity of OsMADS23 by phosphorylation

**Fig 11:**
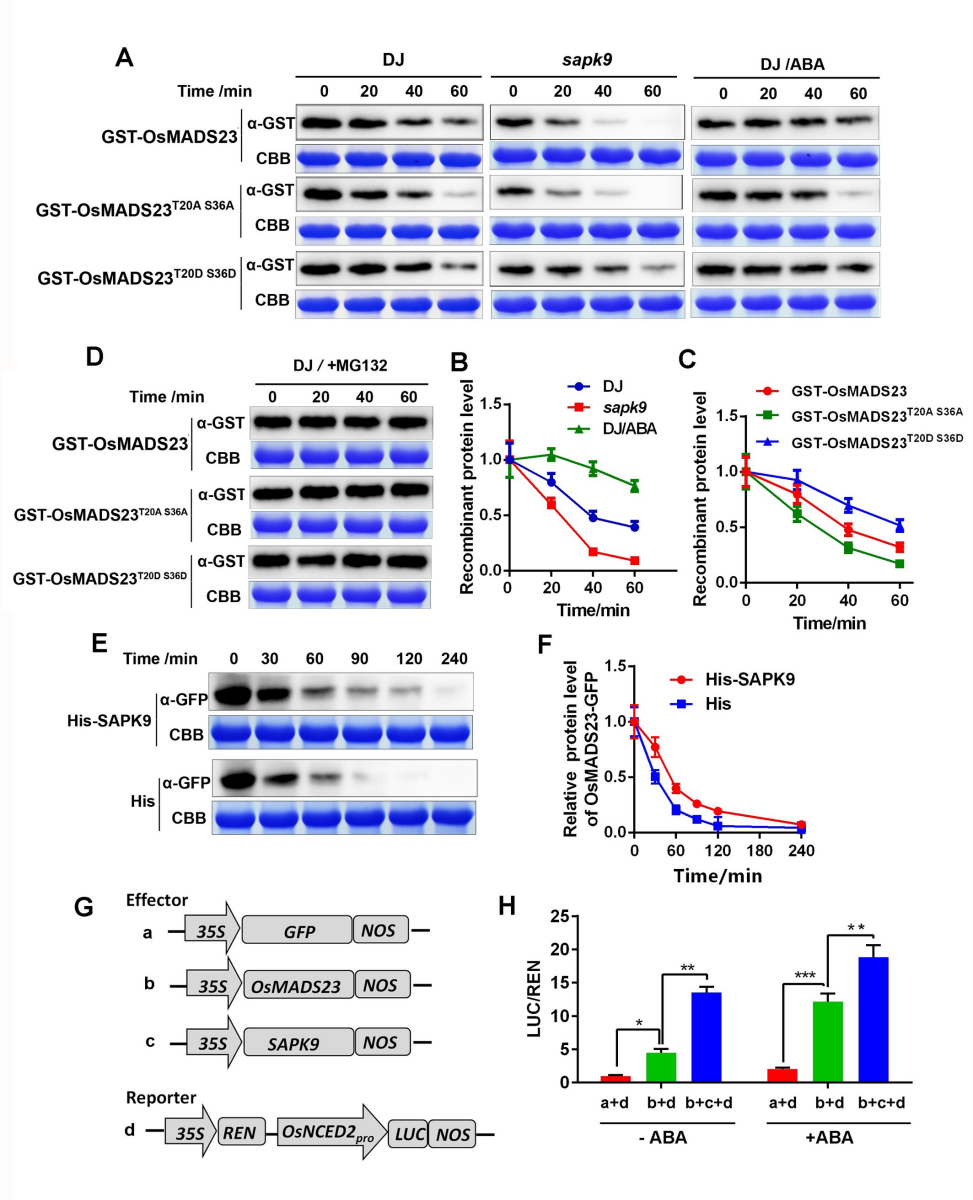
The phosphorylation of OsMADS23 by SAPK9 is required for its stability and transcriptional activity in an ABA-dependent manner. **(A)** Cell-free degradation assays of GST-OsMADS23 or its different mutated versions (GST-OsMADS23^T20A S36A^ and GST-OsMADS23^T20D S36D^) in wild type (WT) or *sapk9* mutant with or without ABA treatment. GST-OsMADS23 and its mutated versions were detected by western blotting using anti-GST antibody. The Coomassie blue–stained ribulose-1,5-bisphosphate carboxylase/oxygenase (Rubisco) large subunit (Rbc L) was used as a loading control. GST-OsMADS23 and its mutated versions were expressed in BL21 (DE3) and purified; an equal amount of each was incubated for different times at 30°C with equal amount protein extracts from leaves of 10-day-old wild-type and *sapk9* plants, with or without 50 μM ABA treatment for 16 h. **(B)** and **(C)** Quantification analysis of the results described in (A**)**. The relative levels of GST-OsMADS23 and its mutated versions in different protein extracts at 0 h were defined as 1. Data represent the means of three independent experiments. **(D)** Cell free degradation of GST-OsMADS23 or its different mutated versions with 50 μM MG132. GST-OsMADS23 and its mutated versions were detected by western blotting using anti-GST antibody. The Coomassie blue-stained ribulose-1,5-bisphosphate carboxylase/oxygenase (Rubisco) large subunit (Rbc L) was used as a loading control. **(E)** Time course of OsMADS23-GFP degradation when the protein extracts from OsMADS23-GFP plants were incubated with His or His-SAPK9. Equal amounts of plant crude extracts were added to equal amounts of the recombinant proteins in the *in vitro* cell-free degradation assays. OsMADS23-GFP was detected by western blotting using anti-GFP antibody. The Coomassie blue-stained ribulose-1,5-bisphosphate carboxylase/oxygenase (Rubisco) large subunit (Rbc L) was used as a loading control. **(F)** Quantification analysis of the results in **(**E**)**. The relative levels of OsMADS23-GFP at 0 h were defined as 1. Data represent the means of three independent experiments. **(G)** Schematic diagram of the constructs used in the transient transactivation assay. **(H)** SAPK9 as well as ABA can increase the transactivation activity of OsMADS23 in *Nicotiana* leaves. One half of the infiltrated leaves were incubated with 50 μM ABA for 16 h. Data represent the means of three independent experiments. *p < 0.05, **p < 0.01 (Student’s *t* test). These cell-free degradation assays were performed in the presence of ATP.

Protein phosphorylation has been indicated to be closely associated with protein turnover and stability [50]. Therefore, we examined the influences of SAPK9-mediated phosphorylation on the stability of OsMADS23 by cell-free protein degradation assays. In comparison with the protein extracts from wild type (DJ), the extracts from *sapk9* mutant obviously promoted the degradation of GST-OsMADS23 (Fig 11A and 11B). Notably, the mimicked phosphorylation (GST-OsMADS23^T20D S36D^) significantly stabilized the protein when incubated with the wild-type protein extracts, while the mimicked dephosphorylation (GST-OsMADS23^T20A S36A^) promoted its degradation (Fig 11A and 11C). In addition, when incubated with protein extracts from *sapk9* mutant, GST-OsMADS23^T20D S36D^ was more stable than GST-OsMADS23 or GST-OsMADS23^T20A S36A^ (Fig 11A). Similar results were obtained when GST-OsMADS23 or its derivatives was incubated with the protein extracts without ATP (S12A and S12B Fig). This result was further confirmed by the increased stability of GST-OsMADS23 when incubated with the protein extracts from *N*. *benthamiana* transiently expressing SAPK9-3×FLAG (S12C Fig). The degradation rates of the recombinant protein GST-OsMADS23 and its derivatives became much slower when added MG132, a 26S proteasome inhibitor (Fig 11D), implying that the protein is under the degradation of the ubiquitin/26S proteasome pathway. Consistently, we found that OsMADS23-GFP degraded more slowly when His-SAPK9 was added in the protein extracts from OsMADS23-GFP plants (Fig 11E and 11F). We further verified the regulatory effect of ABA on OsMADS23 stability. Compared to the mock protein extracts, the extracts from wild type treated with ABA significantly delayed the degradation of GST-OsMADS23 (Fig 11A and 11B), suggesting that ABA can enhance the stability of OsMADS23. This result was confirmed by the increased accumulation of OsMADS23-GFP in OsMADS23-overexpressing plants treated by ABA (S12D Fig). Taken together, the results indicate that the ABA-induced phosphorylation of OsMADS23 mediated by SAPK9 is critical for its stability.

To further investigate whether the SAPK9-mediated phosphorylation on OsMADS23 can increase its transcriptional activity in plants, OsMADS23 was coexpressed with SAPK9 in *N*. *benthamiana* leaves (Fig 11G). We found that, in the presence of SAPK9, the transcriptional activity of OsMADS23 was greatly increased, compared to that of only OsMADS23 as effector (Fig 11H), suggesting that SAPK9 can elevate the transcriptional activity of OsMADS23. Moreover, we further investigated the transcriptional activity of OsMADS23 in the presence of ABA. After ABA treatment, the transcriptional activity of OsMADS23 was significantly enhanced, compared to mock (Fig 11H). These results suggest that OsMADS23 acts in an ABA-dependent manner, and that an upstream activator such as SAPK9 may be required for its activation.

## Discussion

### OsMADS23 functions as a positive regulator to modulate osmotic stress tolerance in rice

With increasing water scarcity and global climate change, drought and high salinity are emerging as a prominent limiting factors for crop production worldwide [51]. It is particularly challenging for the production of rice, which serves as the food for more than half of the population around the world. Therefore, enhancement of osmotic stress tolerance in rice plants is a fundamental issue. Recently, there are several lines of evidence have shown the crucial roles of MADS-box TFs in plant response to environmental cues. In Arabidopsis, AGL21 modulates osmotic stress tolerance by regulating the expression of *ABSCISIC ACID INSENSITIVE5* (*ABI5*) [34]. In rice, OsMADS25 activates the salt escape response by modulating the expression of *Glutathione S-transferase* (*OsGST4*) and *Pyrroline-5-carboxylate Reductase* (*OsP5CR*) [37]. OsMADS57 is required for chilling tolerance [52], and OsMADS27 can promote the salt stress resistance [53] in rice plants. OsMADS23, OsMADS27, and OsMADS57 are targets of miR444 and they can form homodimers or/and heterodimers [54], suggesting that their dimers or multimeric complexes may coordinately control the expression of target genes. This promotes us to explore the potential role of OsMADS23 in plant adaption to abiotic stress. As a fact, our result showed that, in parallel to OsMADS25 and OsMADS27, OsMADS23 also confers osmotic stress tolerance in plants (Figs 2–4 and S4). Thus, in future work, it is of great interest to reveal the genetic basis and molecular mechanism that OsMADS23 coordinates with OsMADS25 and/or OsMADS27 to regulate plant response to osmotic stress.

### OsMADS23 confers osmotic stress tolerance in an ABA-dependent manner

It is well described that ABA modulates plant adaptation to osmotic stress mainly through increasing cellular dehydration tolerance and reducing water loss [55]. The former function was conferred particularly by inducing the expression of dehydration-responsive genes, and the latter trait was closely related to the regulation of stomatal closure [56]. Relevant to above findings, in our study, *OsMADS23*-overexpressing lines increased but its knockout mutant reduced the hypersensitivity to exogenous ABA (Fig 5A–5D). Meanwhile, under drought stress conditions, *OsMADS23*-overexpressing plants had higher expression of ABA-dependent stress-responsive genes than wild type (Fig 5G). In accordance with this, after ABA treatment, the stomata aperture in *OsMADS23*-overexpressing leaves was remarkably reduced (Fig 5F), indicating that *OsMADS23* functions positively in ABA-induced stomatal closure. All the above results indicate that drought and salt stress tolerance conferred by OsMADS23 is dependent on ABA, which is consistent with previous reports that osmotic stress conditions can trigger the ABA-dependent signaling pathway [51].

### OsMADS23 promotes ABA biosynthesis by activating *OsNCEDs* directly

NCED has been shown to contribute to increased ABA levels and abiotic stress tolerance in plants [20]. NCED genes such as *VP14* [21], *SgNCED* [57], *AtNCED3* [27] and *CsNCED3* [58] were previously reported to alter water stress sensitivity if expression was inhibited or promoted in plants. In rice, five members of the NCED family (OsNCED1-5) have been characterized [59,60]. Of those, *OsNCED3*, *OsNCED4*, and *OsNCED5* have been suggested to be correlated with stress-induced ABA biosynthesis [59]. Recently, transgenic plants have shown that *OsNCED3* and *OsNCED4* play vital roles in ABA biosynthesis and osmotic stress tolerance [25,45]. *OsNCED2* was shown to play the predominant role in ABA biosynthesis in imbibed rice seeds, and was also induced by osmotic stress [60], hinting that *OsNCED2* might be functionally related to abiotic stress tolerance via ABA biosynthesis. Thus, understanding how *OsNCEDs* are activated in response to osmotic stress is important for the elucidation of the mechanisms that govern plant acclimation to abiotic stress. In our study, as the strong evidence supporting OsMADS23 confers the osmotic stress tolerance through modulating ABA levels in rice, OsMADS23 acted upstream of *OsNCED2*, *OsNCED3* and *OsNCED4*, and positively regulated their expression (Figs 6 and 7). More importantly, in parallel to *osmads23* mutant, *osnced2* mutants had reduced ABA accumulation and increased sensitivity to drought and oxidative stress (Fig 8). In addition, proline synthetic gene *OsP5CR* was found to be directly targeted by OsMADS23 (Fig 7), which is consistent with that ABA-induced salt tolerance might be associated with the expression of *OsP5CR* [46]. The results further support that OsMADS23 regulates osmotic stress tolerance in an ABA-dependent manner.

### OsMADS23 needs phosphorylation for its stability and transcriptional activity

Some of the bZIP TFs have been shown to require phosphorylation for their transcriptional activation. It is well known that activated SnRK2s further pass the signals to the targets in the post-translational level, majorly through protein phosphorylation on their conserved motifs like R-X-X-S/T [8]. Cotransformation of OsbZIP46 with SAPK2, SAPK6, or SAPK9 into rice protoplasts can significantly enhance its transactivation activity [15,16,61]. In our study, OsMADS23 was found to physically interact with SAPK9 and be phosphorylated by SAPK9 (Figs 9 and 10). SAPK9-mediated phosphorylation on OsMADS23 significantly enhanced its stability and transcriptional activity, and ABA treatment further drastically increased its activity (Fig 11), indicating that SAPK9 could activate OsMADS23 by phosphorylation, in an ABA-dependent manner. Previous study showed that overexpression of *SAPK9* in rice increased plant tolerance to drought stress by regulating stomatal closure [48], indicating the crucial roles of SAPK9 in osmotic stress tolerance. Consistent with this, our results showed that OsMADS23 positively regulated the ABA-induced stomatal closure (Fig 5F), which further supports our speculations that OsMADS23 phosphorylation mediated by SAPK9 is required for its roles in the osmotic stress tolerance. However, GST-OsMADS23^T20A S36A^ that had both of the two amino acid substitutions in the potential phosphorylation sites was still found to be weakly phosphorylated by SAPK9 *in vitro* assay (Fig 10A), hinting that there are minor phosphorylation sites still in the dark. Thus, the minor phosphorylation sites recognized by SAPK9 remain to be revealed in the future.

## Conclusion

**Fig 12:**
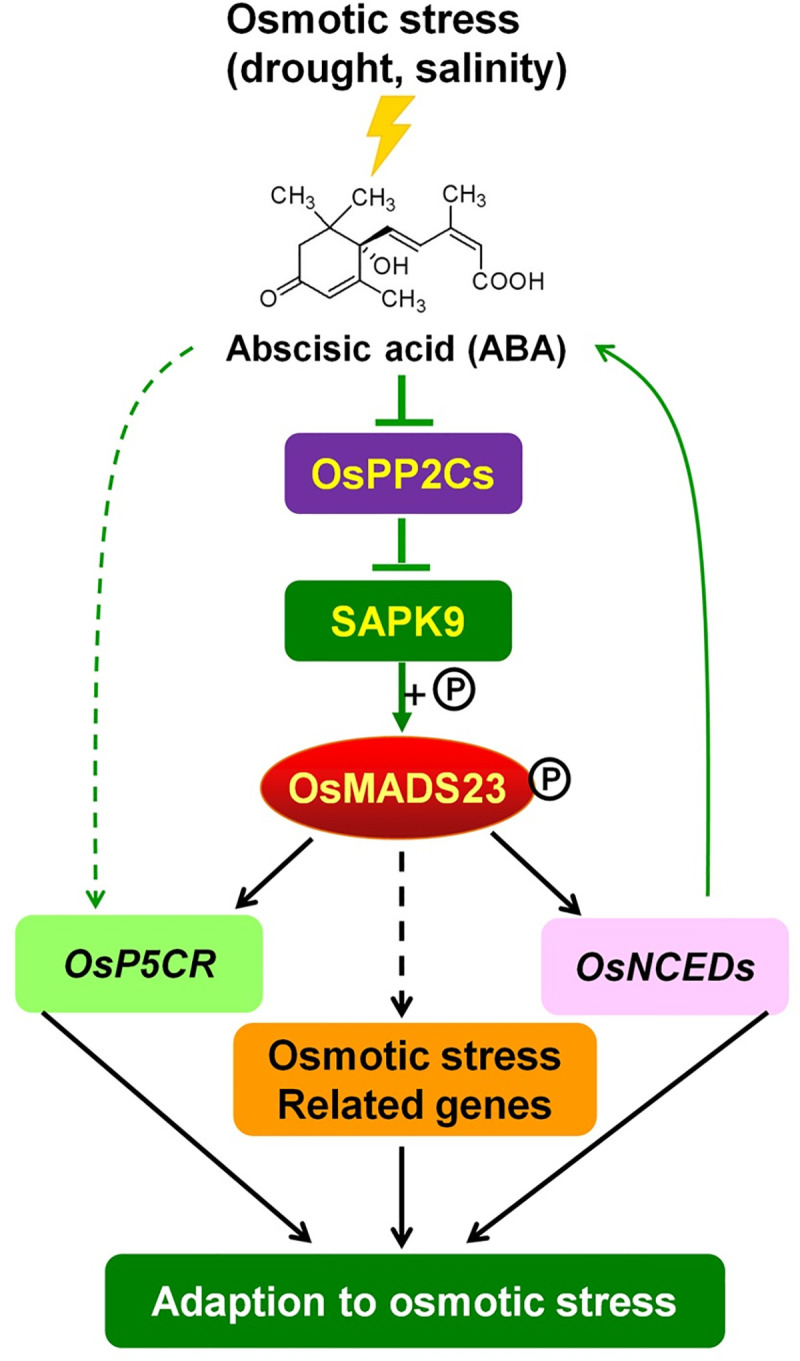
Working model of OsMADS23 conferring the osmotic stress tolerance via the ABA signaling in rice. ABA, which is induced by abiotic stress such as drought or salt stress, inhibits the activity of OsPP2Cs to release the kinase activity of SAPK9 for further activation of OsMADS23 through phosphorylation. OsMADS23 could promote the ABA levels through directly activating the expression of *OsNCEDs*. Meanwhile, OsMADS23 also enhanced proline content by targeting proline synthesis gene *OsP5CR*. The circled “P” indicates phosphorylation (+P).

In this work, we found that OsMADS23 functions as a positive regulator to improve the tolerance to drought and salt stress by directly activating the ABA synthetic genes *OsNCED*s and proline synthetic gene *OsP5CR* in rice plants. More importantly, SAPK9, an upstream kinase in ABA signaling, was shown to phosphorylate OsMADS23 and enhance its stability and transcriptional activity. Activated OsMADS23 could enhance ABA levels and proline accumulation, and thus improve osmotic stress tolerance in plants (Fig 12). These results establish a regulatory network of OsMADS23 in the ABA signaling pathway, suggesting that OsMADS23 might act as a promising candidate gene for engineering osmotic stress resistance in rice.

## Materials and methods

### Plant materials and growth conditions

For generating *OsMADS23*-overexpressing lines, the full-length coding sequence of *OsMADS23* was cloned into pCAMBIA1301, driven by *35S* promoter and then transformed into rice cultivar Nipponbare (Nip, *Oryza sativa* L. ssp. japonica) by *Agrobacterium tumefaciens*-mediated transformation [62], and seeds of homozygous plants were used. T-DNA insertion mutants *osmads23-1* (RMD_ITL-03Z11CH52_ULB3) and *osmads23-2* (RMD_TTosL-03Z11JN10_TosRS) in Zhonghua 11 (Z11) background were from RMD mutant database [63]. The *osnced2* mutants (Z11 background) were generated by CRISPR-Cas9 system [64]. The *sapk9* mutant (PFG_3A-60717.L) was obtained from the T-DNA insertional population in *Oryza sativa* ssp. *japonica* cv. Dongjin (DJ) [65]. The homozygous mutants were identified by PCR or sequencing analysis. Plants were grown in the growth chamber or greenhouse with a 14 h light (30°C) /10 h dark (25°C) cycle (300 μmol photons m^−2^ s^−1^) with 60% humidity. Primers are given in S2 Table.

### Drought- and salt-tolerance assays

Seedlings were grown on half-strength MS medium supplemented with 150 mM NaCl, 20% PEG6000 or 2 μM MV. For testing the drought or salt stress tolerance in soil, 4-week-old rice plants were used. Then water was withheld from plants for drought stress treatment, or plants were irrigated every 3 day with 300 mM NaCl solution for salt stress (the control plants were irrigated with water), until the leaves of wild type became completely wilted. The plants were then rewatered and the number of surviving plants was counted.

### Water loss rate measurement

For the water loss rates, the leaves of 70-day-old plants were detached and placed at room temperature. The fresh weight of detached leaves was monitored at the indicated time points. Water loss was calculated from the decrease in the fresh weight compared with time zero. The average water loss rate was calculated from three independent experiments.

### Stomatal observation

Full expanded young leaves of 7-day-old plants were detached and treated with 50 μM ABA treatment in MES-KCl buffer (50 mM KCl, 10 mM MES-KOH, pH 6.15) for 2 h. Stomatal closure was detected by Hitachi SU3500 scanning electron microscope with a -40°C cool stage. Three hundred stomata of each line were observed and the completely open, partially open, and completely closed stomata were analyzed as described previously [44].

### Measurement of ABA content

ABA extraction and quantification was performed as described previously [66]. Briefly, 50 mg of leaf samples of 3-week-old seedlings were freeze-dried and extracted twice with 0.5 ml of plant hormone extraction buffer (methanol: water: glacial acetic acid, 80: 20: 1, v/v/v) supplemented with 2 ng of ABA-d6 internal standards. Quantification was performed in an ABI 4000 Q-Trap (Applied Biosystems).

### Physiological analysis of stress-associated indicators

MDA content was measured as previously described [67]. For ROS assays, nitroblue tetrazolium (NBT) staining was used to detect O_2_^-^ and 3’-diaminobenzidine (DAB) staining for H_2_O_2_, as described previously [68]. H_2_O_2_ quantification was performed as described previously [69]. Total chlorophyll content was determined as described previously [70]. Free proline content was measured using the reported method [71]. The activities of superoxide dismutase (SOD) and catalase (CAT) were determined as described previously [72].

### RNA extraction and quantitative PCR analysis

Total RNA was extracted using TRIzol reagent (Takara). Quantitative PCR analysis was performed with rice *β*-*actin* as the internal control. Relative changes in gene expression levels were quantitated based on three biological replicates via the 2^-ΔΔCt^ method [73]. Primers used for expression analysis are listed in S2 Table.

### Transient transactivation assay

We used pGreenII cloning vectors to construct transactivation plasmids for dual-luciferase assays in protoplasts [74]. The full-length coding sequence of *OsMADS23* was cloned into pGreenII 62-SK to act as the effector; the promoter fragment of target gene was cloned into pGreenII 0800-LUC as the reporter, and the Renilla luciferase (*REN*) gene driven by *35S* promoter in pGreenII 0800-LUC was used as an internal control. Rice protoplasts were prepared and then transfected using a polyethylene glycolcalcium-mediated method followed by a 20-h incubation to allow transient expression [75]. Firefly LUC and REN activities were measured with a dual-luciferase reporter assay kit (Promega). Additionally, To test the transient transactivation, pGreenII 0800-GUS, in which the luciferase (*LUC*) gene in pGreenII 0800-LUC was substituted with *GUS*, was used. These constructs were individually transformed into the *A*. *tumefaciens* strain GV3101. *Agrobacterium* strains carrying the reporter or effector constructs were coinfiltrated into 3-week-old *N*. *benthamiana* leaves and incubated for 2–3 days. GUS histochemical staining was detected as described previously [44]. Primers used for these constructs are listed in S2 Table.

### Electrophoretic mobility shift assay (EMSA)

The full-length coding sequence of *OsMADS23* was fused in-frame with His in pCold-TF and expressed in *Escherichia coli* BL21 (DE3) cells, and the recombinant His-OsMADS23 was purified. Oligonucleotide probes containing CArG-box motifs were synthesized and labeled with using a Biotin 3’ End DNA Labeling Kit (Thermo), and EMSA was performed using a Lightshift Chemiluminescent EMSA kit (Thermo) according to the manufacturer’s instructions. Probes used are listed in S2 Table.

### Yeast two-hybrid assay

This assay was performed as described previously [76]. The full-length coding sequence of *SAPK9* was cloned into the pGBKT7 (binding domain [BD]) vector, and *OsMADS23* into pGADT7 (activation domain [AD]) vector. These resulting constructs were cotransformed into the yeast strain Y2H Gold (Clontech) for two-hybrid assay. Interaction was determined by growth assay on defined media (SD/-Ade-His-Leu-Trp) in the presence of aba and X-α-gal. Primers for these constructs are listed in S2 Table.

### GST pull-down assay

The full-length coding sequence of *OsMADS23* was cloned into pGEX-4T-1 and transformed into DE3 to produce the GST-OsMADS23, and *SAPK9* into pET-32a (+) to produce the His-SAPK9 (for primers, see S2 Table). For pull-down assay, 0.5 mg of GST-OsMADS23 or GST was incubated with GST Bind Resin at 4°C for 2 h, and then 0.5 mg of purified His-SAPK9 was added. The incubation continued for another 2 h, and the beads were washed with pull-down buffer for three times. The bounded proteins were finally eluted, and the pulled down protein was separated on 10% SDS-PAGE gel and were analyzed by western blot with the anti-His antibody (Proteintech).

### Bimolecular fluorescence complementation (BiFC) assay

BiFC vectors pFGC-nYFP and pFGC-cYFP [77] were used. The full-length coding sequence of *OsMADS23* was cloned into pFGC-cYFP, resulting in OsMADS23-cYFP, and *SAPK9* into pFGC-nYFP to generate SAPK9-nYFP (for primers, see S2 Table). OsMADS23-cYFP and SAPK9-nYFP were cotransformed into rice protoplasts by PEG-mediated transformation method [75]. The nYFP and cYFP empty vectors were used as the negative controls for the assay. Fluorescence signals were visualized using a Leica SP8 confocal microscope.

### Coimmunoprecipitation (CoIP) assay

To detect whether OsMADS23 can interact with SAPK9 *in vivo*, constructs OsMADS23-GFP and SAPK9-3×FLAG were individually transformed *Agrobacterium* strain GV3101, and then coinjected into leaves of 3-week-old seedlings of *N*. *benthamiana*. After transiently coexpressed for 2–3 days, the leaves were collected and total protein was extracted with protein extraction buffer (50 mM Tris, pH 7.4, 150 mM NaCl, 1% NP-40, 0.25% sodium dexycholate, 0.1% sodium fluoride, 0.5 mM EDTA, 1× Protease Inhibitor Cocktail and and 0.2 mM PMSF). The extracted protein was incubated with pre-washed anti-GFP Protein A/G agarose beads (Abmart) overnight at 4°C, and the beads were washed three times using 2×protein extraction buffer and boiled in 1×SDS loading buffer for analysis. The anti-GFP (Proteintech) and anti-FLAG (Proteintech) antibodies were as used to test the CoIP results.

### *In vitro* kinase assay

*In vitro* kinase assays were performed as previously described [49]. Briefly, the native or mutated version of GST-OsMADS23 was coexpressed with His or His-SAPK9 in *E*. *coli* BL21 (DE3), and the native or mutated version of His-SAPK9 was coexpressed with GST-OsMADS23 in DE3. Proteins were purified using the GST Bind Resin (Proteintech), and then separated by SDS-PAGE with or without 50 mM Phos-tag (APE×BIO). The signals were detected with anti-GST and -His antibodies (Proteintech).

### *In vivo* phosphorylation assay

To detect OsMADS23 phosphorylation mediated by SAPK9 *in vivo*, OsMADS23-GFP was transiently coexpressed with 3×FLAG, SAPK9-3×FLAG or SAPK9^S176A^-3×FLAG in 3-week-old *N*. *benthamiana* leaves. The protein was extracted according to the procedures in the CoIP assay (above), and then separated by SDS-PAGE with or without 50 mM Phos-tag (APE×BIO). The signals were detected with anti-GFP and -FLAG antibodies (Proteintech).

### Cell-free degradation assay

Two-week-old seedlings of wild type (DJ) or *sapk9* mutant were used to extract protein. Total protein was extracted in the degradation buffer (25 mM Tris-HCl, pH 7.5, 10 mM NaCl, 10 mM MgCl_2_, 5 mM DTT, and 10 mM ATP). The same amount of extracts was added to the tubes containing equal amount of recombinant proteins and incubated for different times.

### Chromatin immunoprecipitation-quantitative PCR (ChIP-qPCR)

The EpiQuik Plant ChIP Kit (Epigentek) was used for ChIP assays. Ten-day-old seedlings overexpressing OsMADS23-GFP were harvested and fixed in 1% formaldehyde. Chromatin was isolated from 2 g crosslinked leaves. To investigate the effect of ABA on OsMADS23 activity, seedlings overexpressing OsMADS23-GFP were were sprayed with 50 μM ABA. After 16 h, samples were collected. Isolated chromatin was sonicated for DNA fragmentation ranging from 200 to 1000 bp. Subsequently, the DNA/protein complex was immunoprecipitated with anti-GFP antibody (Proteintech). Then the immunoprecipitated DNA was purified with phenol/chloroform after reverse crosslinking and proteinase K treatment. The immunoprecipitated DNA was used for qPCR analysis. The primers used were listed in S2 Table.

## Supporting information

S1 Fig *osmads23* mutants exhibited repressed growth. **(A)** Schematic diagram indicating the T-DNA insertion sites in genomic region in *osmads23* mutants. **(B)** Molecular identification of *osmads23* mutants by PCR analysis. **(C)** Growth of *osmads23* mutants (M1 and M2) and wild type (Z11) in half-strength MS medium for 7 days. Scale bars, 3 cm. **(D)** Quantification of shoot length of the results described in (C). Error bars indicate SD with biological triplicates (*n* = 3, each replicate containing 20 plants). **(E)** Schematic diagram indicating the T-DNA insertion caused the DNA deletion in genomic region in *osmads23* mutants (M1 and M2). The red dot-line boxes represent DNA deletion. The significant difference between *osmads23* mutants and wild type was determined by Student’s *t* test. *p < 0.05, **p < 0.01 or ***p < 0.001. Three independent experiments were performed.
(TIF)

S2 Fig Phenotypes of multiple transgenic lines overexpressing *OsMADS23* (OE1-OE20). Uniformly germinated seeds were grown in half-strength MS medium for 7 days. Scar bars, 2 cm.
(TIF)

S3 Fig Transcription profiles of *OsMADS23* in response to various environmental stresses by quantitative PCR analysis. *OsMADS23* expression in the roots of 10-day-old seedlings during the time course after 150 mM NaCl **(A)**, 20% PEG6000 **(B)** or 150 mM mannitol **(C)** treatments. Data represent the means of three independent experiments.
(TIF)

S4 Fig Performance of *osmads23*-1 mutant and *OsMADS23*-overexpressing lines exposed to osmotic stress in half-strength MS medium. **(A)** Performance of wild type (Nip) and *OsMDS23* overexpression plants (OE13 and OE14) exposed to 150 mM NaCl and 20% PEG6000, respectively, for 7 days. Scale bars, 1 cm. **(B) and (C)** Shoot length and primary root (PR) length in plants exposed to osmotic stress for 7 days, respectively. **(D)** Performance of wild type (Z11) and *osmads23-1* (M1) exposed to osmotic stress for 7 days. Scale bars, 2 cm. **(E)** Shoot length in *osmads23-1* and wild type exposed to osmotic stress for 7 days. **(F)** Phenotypes of wild type and overexpression lines exposed to osmotic stress for 14 days. Scale bars, 2 cm. **(G)** and **(H)** Shoot length and chlorophyll content in plants exposed to osmotic stress for 14 days, respectively. PR, primary root; Chl, chlorophyll. In B, C, E and G, Error bars indicate SD with biological triplicates (*n* = 3, each replicate containing 20 plants). In H, Error bars indicate SD with biological triplicates (*n* = 3, each replicate containing 3 plants). The significant difference between *OsMADS23*-overexpressing lines and wild type was determined by Student’s *t* test. *p < 0.05, **p < 0.01 or ***p < 0.001. All data displayed as a mean ± SD. Three independent experiments were performed.
(TIF)

S5 Fig ROS accumulation in *OsMADS23*-overexpressing lines and *osmads23-1* mutant. **(A)** and **(B)** H_2_O_2_ levels in *OsMADS23*-overexpressing lines (OE13 and OE14) and *osmads23-1* mutant (M1) exposed to drought stress for 5 days, respectively. **(C)** and **(D)** NBT staining in the leaves of *OsMADS23*-overexpressing lines and *osmads23-1* mutant exposed to drought stress for 5 days, respectively. **(E)** Expression of ROS-scavenging genes in plants under normal conditions. **(F)** and **(G)** Activities of SOD and CAT in plants exposed to drought stress for 5 days, respectively. **(H)** and **(I)** Content of proline and MDA in plants exposed to drought stress for 5 days. The significant difference between *OsMADS23*-overexpressing lines or *osmads23-1* mutant and their corresponding wild-type plants was determined by Student’s *t* test. *p < 0.05, **p < 0.01. All data displayed as a mean ± SD. Three independent experiments were performed (n = 3 plants per genotype in each independent experiment).
(TIF)

S6 Fig OsMADS23 mediates ABA sensitivity in seeds germination and plant growth. **(A)** Images of seed germination of *OsMADS23*-overexpressing plants (OE13 and OE14) or *osmads23-1* mutant (M1) and their corresponding wild type (Nip or Z11) on half-strength MS medium without or with ABA for 4 days, respectively. **(B)** Seed germination rates of the results described in **A**. Error bars indicate SD with biological triplicates (*n* = 3, each replicate containing 50 seeds). **(C-F)** Shoot and primary root length in different genotypes with or without ABA for 4 days, respectively. Error bars indicate SD with biological triplicates (*n* = 3, each replicate containing 30 plants). **(G)** Expression of ABA-responsive genes in plants under normal conditions. Error bars indicate SD with biological triplicates. *p < 0.05, **p < 0.01 or ***p < 0.001 (Student’s *t* test). Three independent experiments were performed.
(TIF)

S7 Fig CRISPR-Cas9-mediated target mutagenesis of *OsNCED2*. **(A)** Frameshift mutations of *OsNCED2* leads to early termination of protein translation, resulting truncated proteins. **(B)** Expression profile of *OsNCED2* in various tissues by quantitative PCR analysis. Error bars indicate SD with biological triplicates.
(TIF)

S8 Fig Subcellular localization of OsMADS23 in the epidermal cell of *Nicotiana* *benthamiana* leaves. (TIF)

S9 Fig Yeast two-hybrid assays of OsMADS23 and SAPK8 or SAPK10. SD, synthetic dropout medium. DDO, SD/-Leu-Trp. QDO, SD/-Ade-His-Leu-Trp.
(TIF)

S10 Fig R-X-X-S/T and R-Q-X-S/T motifs in OsMADS23 protein sequence. (TIF)

S11 Fig *sapk9* is a loss-of-function mutant. **(A)** Schematic diagram indicating the T-DNA insertion site in genomic region in *sapk9* mutant. **(B)** Molecular identification of *sapk9* mutant by PCR analysis. **(C)** Transcript levels of *SAPK9* in wild type (DJ) and *apk9* mutant by quantitative PCR analysis. Error bars indicate SD with biological triplicates.
(TIF)

S12 Fig The effects of SAPK9 or ABA on the stability of OsMADS23. **(A)** The cell degradation assay of GST-OsMADS23 and its different mutated versions in the protein extracts (without ATP) from DJ (wild type) and *sapk9* mutant. **(B)** Quantification analysis of the results described in (**A)**. The relative levels of GST-OsMADS23 and its mutated versions in different protein extracts at 0 h were defined as 1. Data represent the means of three independent experiments. **(C)** The cell degradation assay of GST-OsMADS23 and its different mutated versions in the protein extracts from *Nicotiana* *benthamiana* leaves transiently overexpressing SAPK-3×FLAG or 3×FLAG alone. **(D)** OsMADS23 degradation in protein extracts from the OsMADS23-GFP plants treated with or without ABA. Plants were treated with 50 μM ABA for 24 h.
(TIF)

S1 Table Agronomic traits of *OsMADS23*-overexpressing lines and wild type (Nip). (DOCX)

S2 Table Primer sequences used for this study. (DOC)
