# Supplementary figures and images for: OsMADS23 phosphorylated by SAPK9 confers drought and salt tolerance by regulating ABA biosynthesis in rice
Source: PLoS Genet. 2021 Aug 3;17(8):e1009699. doi: 10.1371/journal.pgen.1009699 (PMC8363014; doi:10.1371/journal.pgen.1009699)

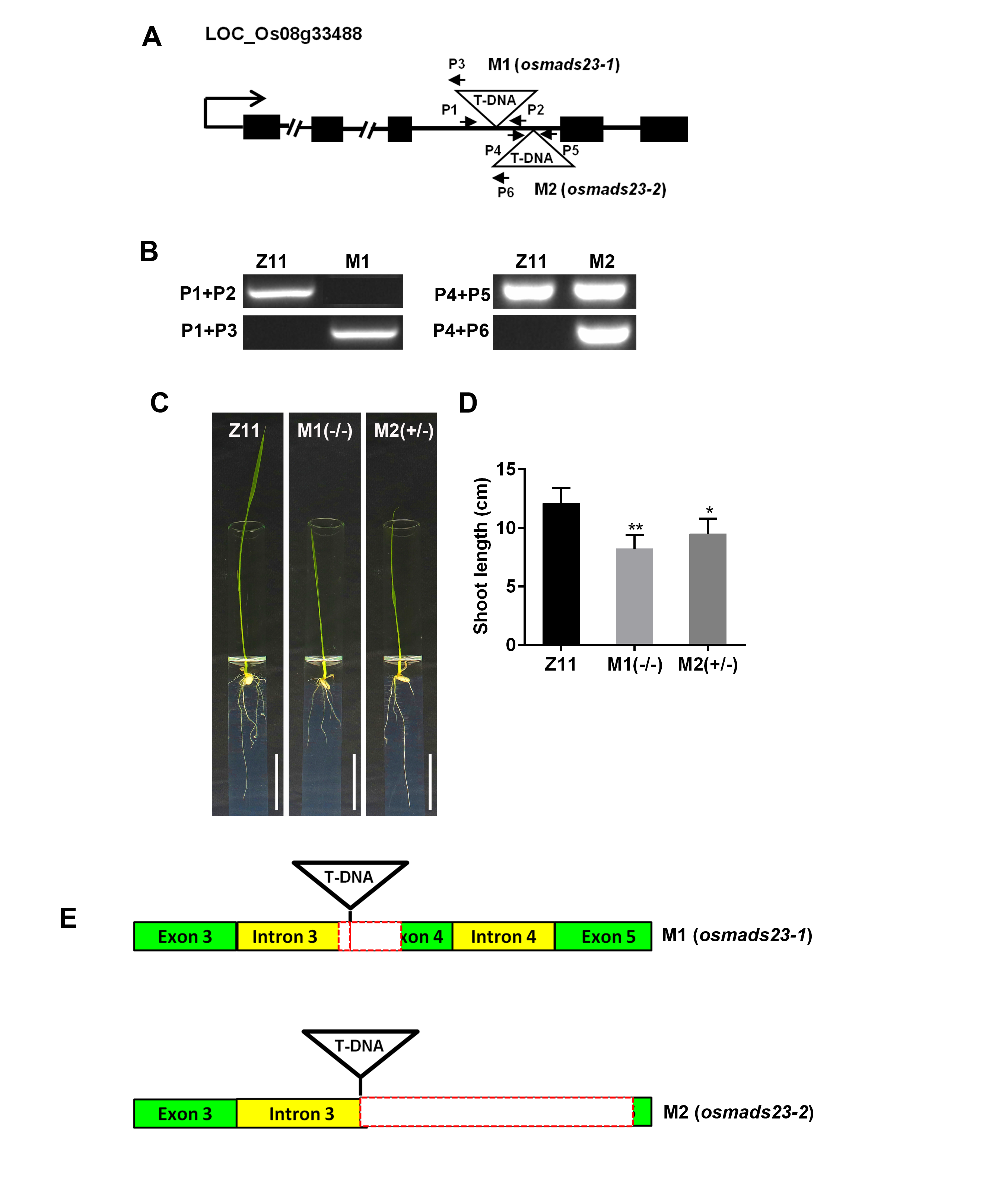

Supplement: S1 Fig — (A) Schematic diagram indicating the T-DNA insertion sites in genomic region in osmads23 mutants. (B) Molecular identification of osmads23 mutants by PCR analysis. (C) Growth of osmads23 mutants (M1 and M2) and wild type (Z11) in half-strength MS medium for 7 days. Scale bars, 3 cm. (D) Quantification of shoot length of the results described in (C). Error bars indicate SD with biological triplicates (n = 3, each replicate containing 20 plants). (E) Schematic diagram indicating the T-DNA insertion caused the DNA deletion in genomic region in osmads23 mutants (M1 and M2). The red dot-line boxes represent DNA deletion. The significant difference between osmads23 mutants and wild type was determined by Student’s t test. *p < 0.05, **p < 0.01 or ***p < 0.001. Three independent experiments were performed. (TIF) [file pgen.1009699.s001.tif]

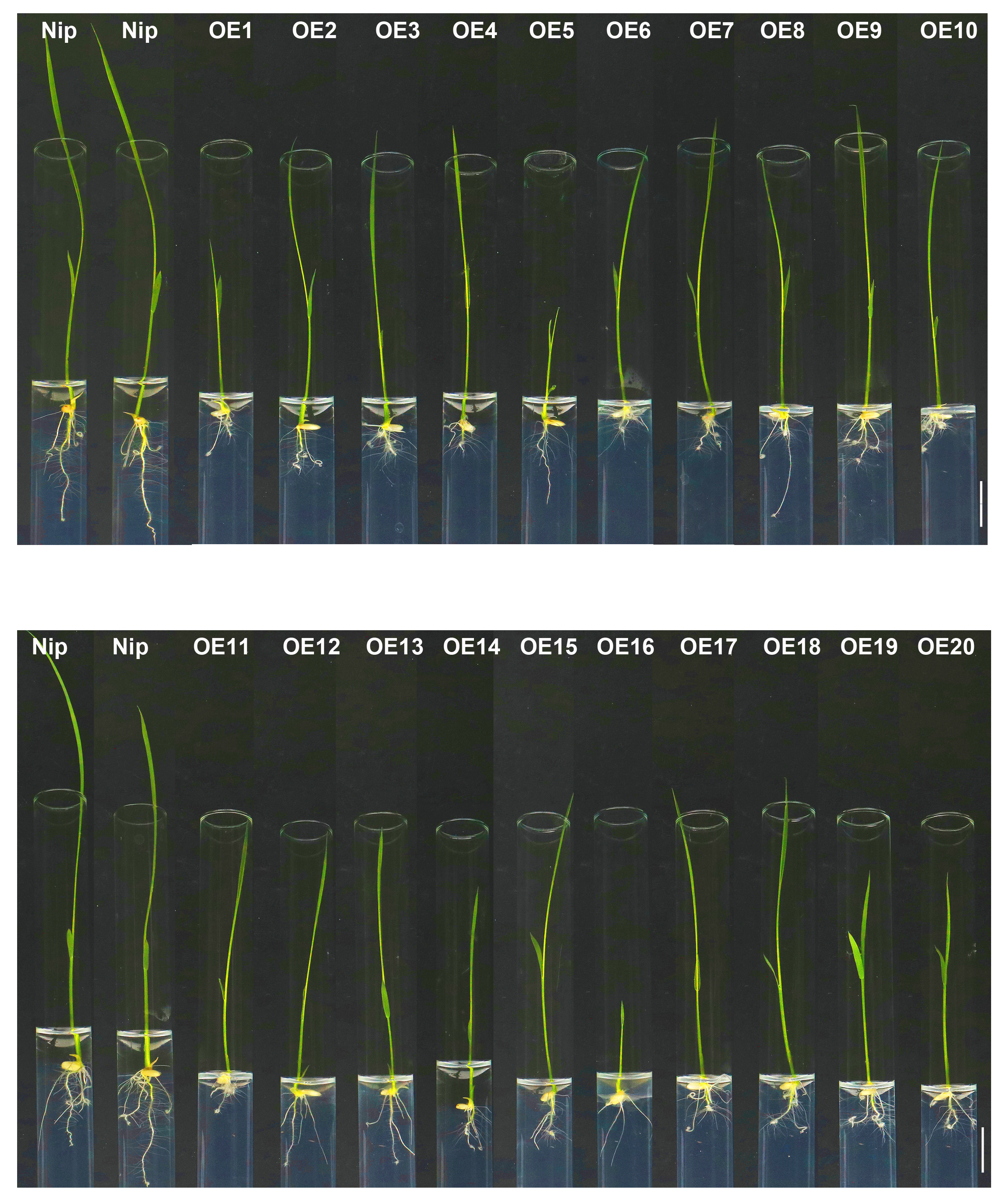

Supplement: S2 Fig — Uniformly germinated seeds were grown in half-strength MS medium for 7 days. Scar bars, 2 cm. (TIF) [file pgen.1009699.s002.tif]

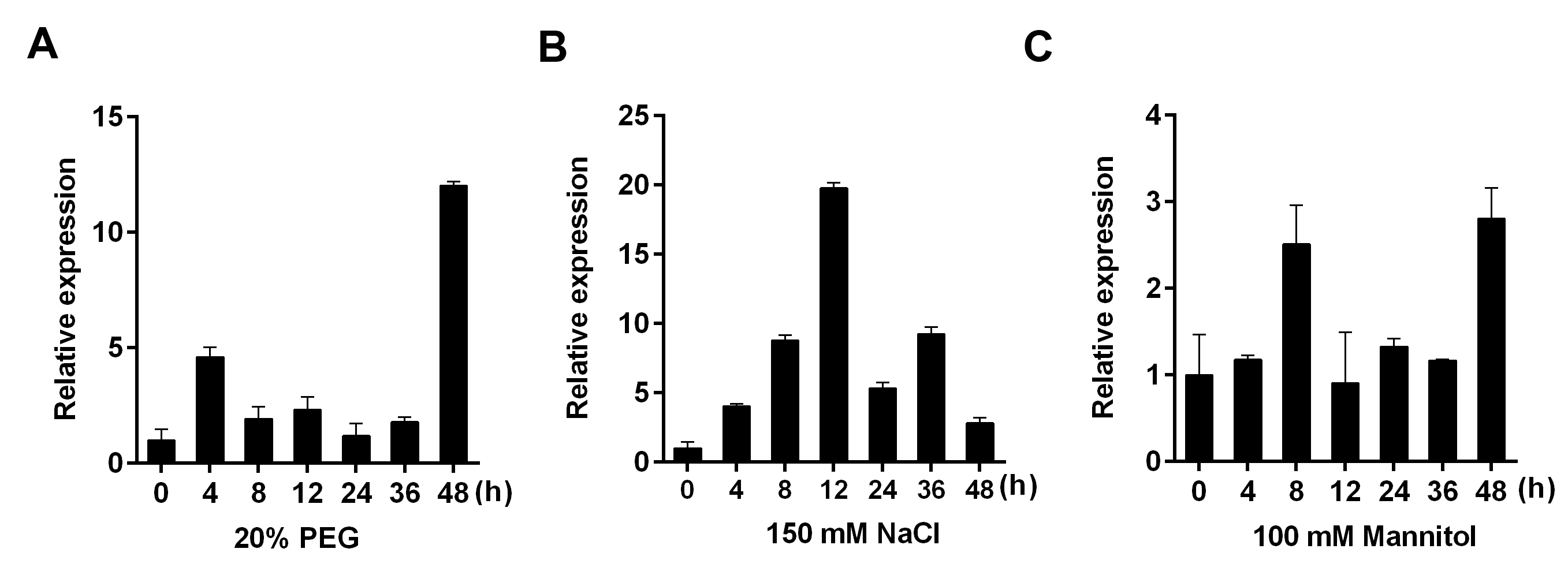

Supplement: S3 Fig — OsMADS23 expression in the roots of 10-day-old seedlings during the time course after 150 mM NaCl (A), 20% PEG6000 (B) or 150 mM mannitol (C) treatments. Data represent the means of three independent experiments. (TIF) [file pgen.1009699.s003.tif]

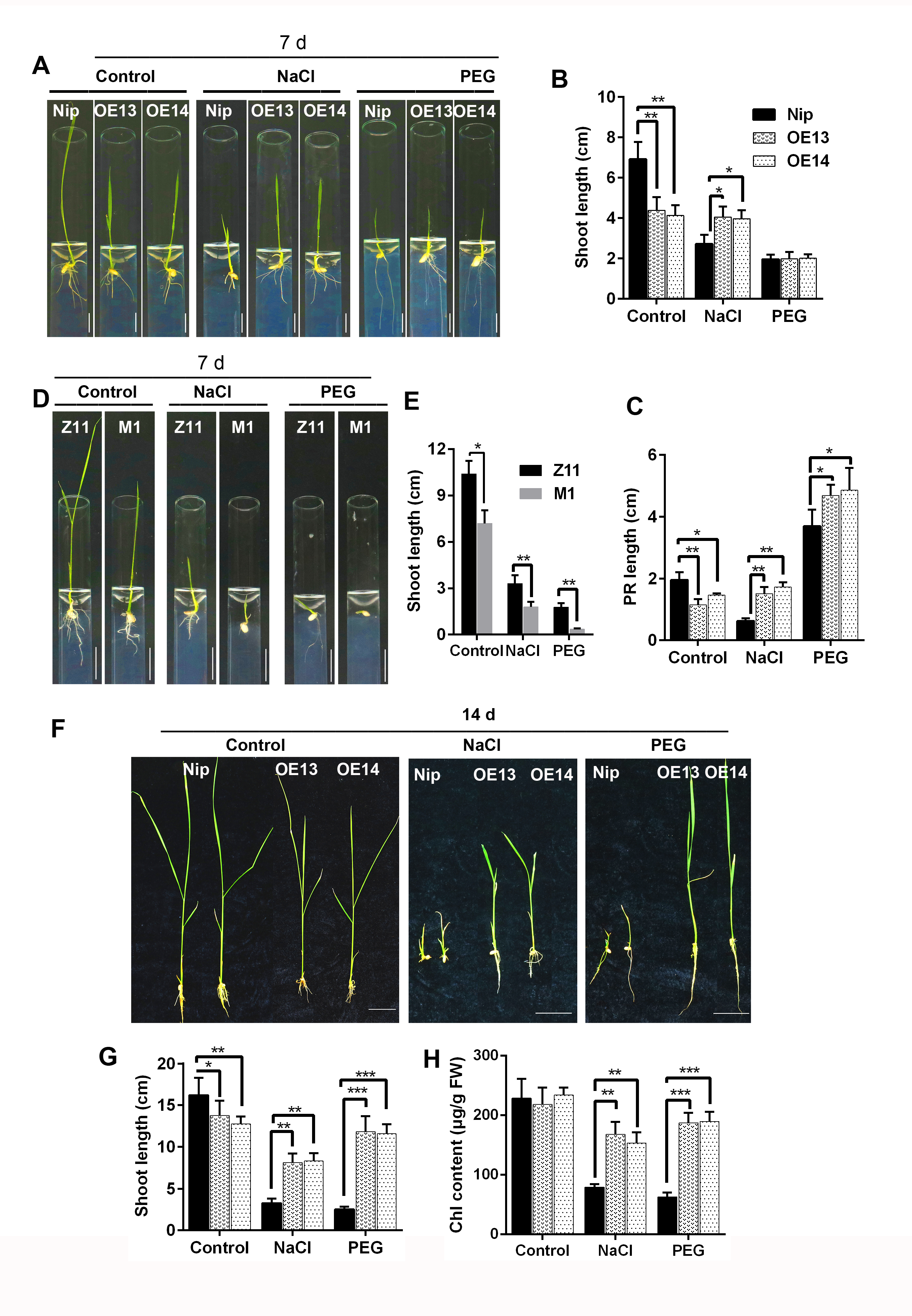

Supplement: S4 Fig — (A) Performance of wild type (Nip) and OsMDS23 overexpression plants (OE13 and OE14) exposed to 150 mM NaCl and 20% PEG6000, respectively, for 7 days. Scale bars, 1 cm. (B) and (C) Shoot length and primary root (PR) length in plants exposed to osmotic stress for 7 days, respectively. (D) Performance of wild type (Z11) and osmads23-1 (M1) exposed to osmotic stress for 7 days. Scale bars, 2 cm. (E) Shoot length in osmads23-1 and wild type exposed to osmotic stress for 7 days. (F) Phenotypes of wild type and overexpression lines exposed to osmotic stress for 14 days. Scale bars, 2 cm. (G) and (H) Shoot length and chlorophyll content in plants exposed to osmotic stress for 14 days, respectively. PR, primary root; Chl, chlorophyll. In B, C, E and G, Error bars indicate SD with biological triplicates (n = 3, each replicate containing 20 plants). In H, Error bars indicate SD with biological triplicates (n = 3, each replicate containing 3 plants). The significant difference between OsMADS23-overexpressing lines and wild type was determined by Student’s t test. *p < 0.05, **p < 0.01 or ***p < 0.001. All data displayed as a mean ± SD. Three independent experiments were performed. (TIF) [file pgen.1009699.s004.tif]

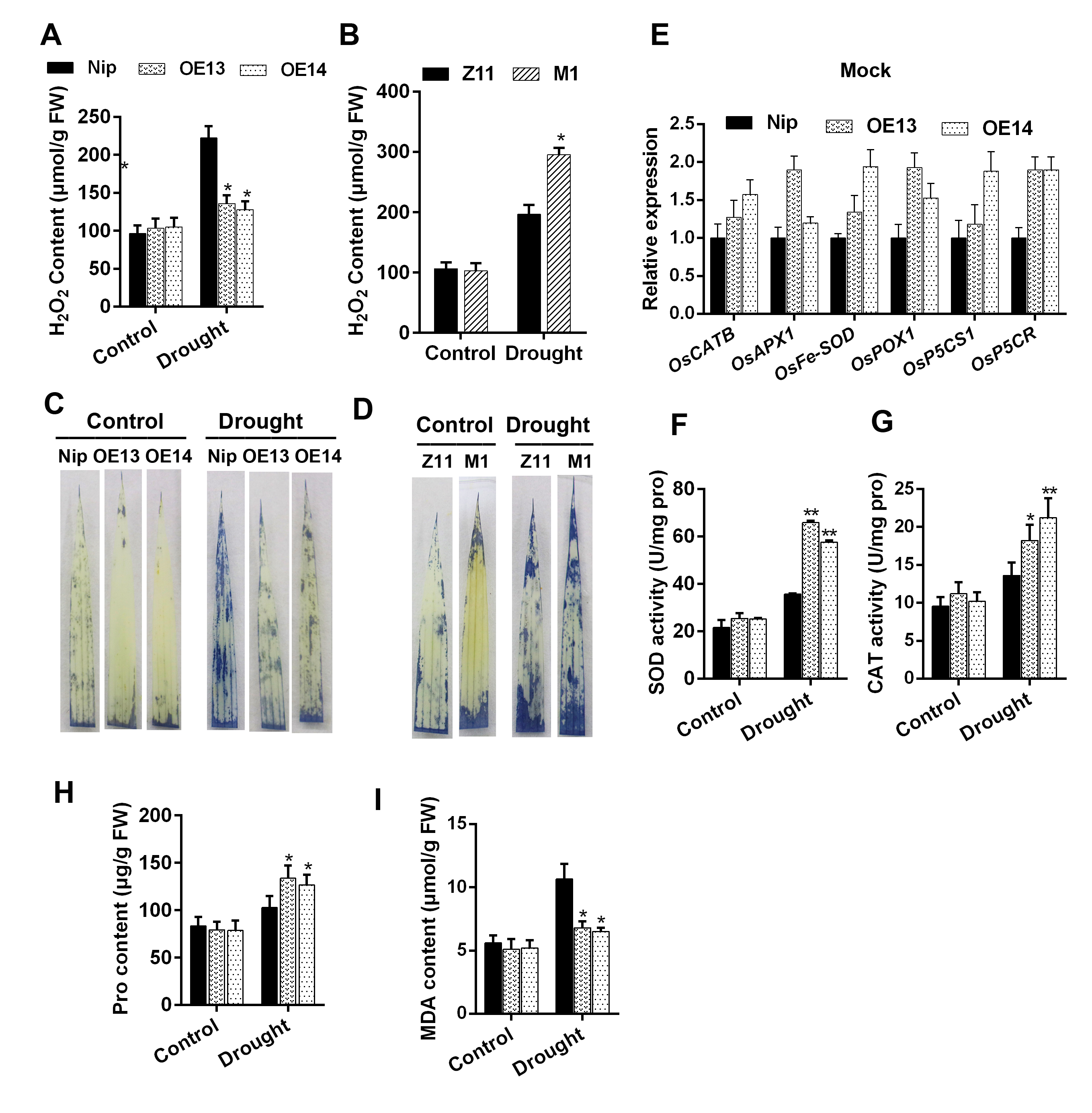

Supplement: S5 Fig — (A) and (B) H2O2 levels in OsMADS23-overexpressing lines (OE13 and OE14) and osmads23-1 mutant (M1) exposed to drought stress for 5 days, respectively. (C) and (D) NBT staining in the leaves of OsMADS23-overexpressing lines and osmads23-1 mutant exposed to drought stress for 5 days, respectively. (E) Expression of ROS-scavenging genes in plants under normal conditions. (F) and (G) Activities of SOD and CAT in plants exposed to drought stress for 5 days, respectively. (H) and (I) Content of proline and MDA in plants exposed to drought stress for 5 days. The significant difference between OsMADS23-overexpressing lines or osmads23-1 mutant and their corresponding wild-type plants was determined by Student’s t test. *p < 0.05, **p < 0.01. All data displayed as a mean ± SD. Three independent experiments were performed (n = 3 plants per genotype in each independent experiment). (TIF) [file pgen.1009699.s005.tif]

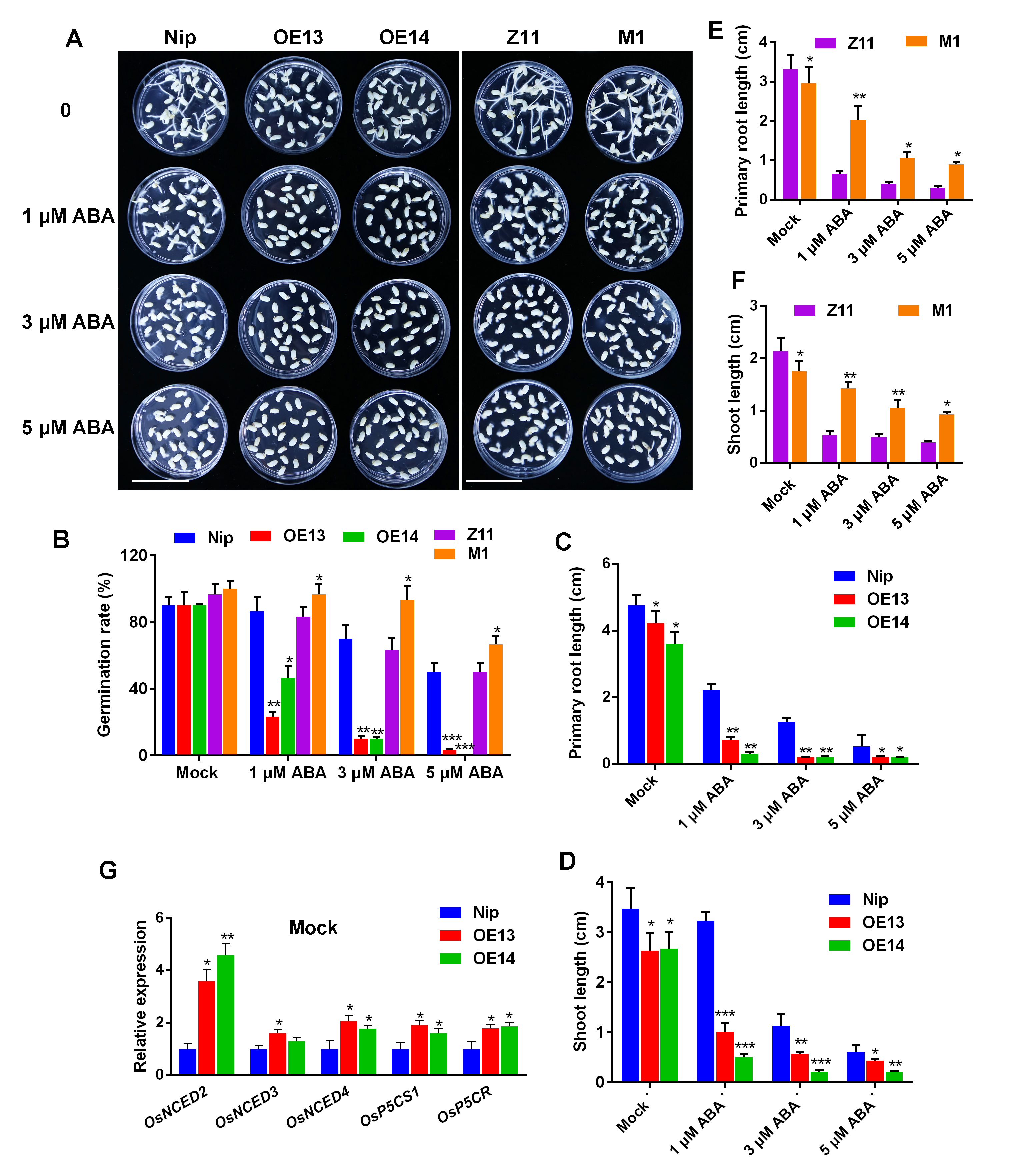

Supplement: S6 Fig — (A) Images of seed germination of OsMADS23-overexpressing plants (OE13 and OE14) or osmads23-1 mutant (M1) and their corresponding wild type (Nip or Z11) on half-strength MS medium without or with ABA for 4 days, respectively. (B) Seed germination rates of the results described in A. Error bars indicate SD with biological triplicates (n = 3, each replicate containing 50 seeds). (C-F) Shoot and primary root length in different genotypes with or without ABA for 4 days, respectively. Error bars indicate SD with biological triplicates (n = 3, each replicate containing 30 plants). (G) Expression of ABA-responsive genes in plants under normal conditions. Error bars indicate SD with biological triplicates. *p < 0.05, **p < 0.01 or ***p < 0.001 (Student’s t test). Three independent experiments were performed. (TIF) [file pgen.1009699.s006.tif]

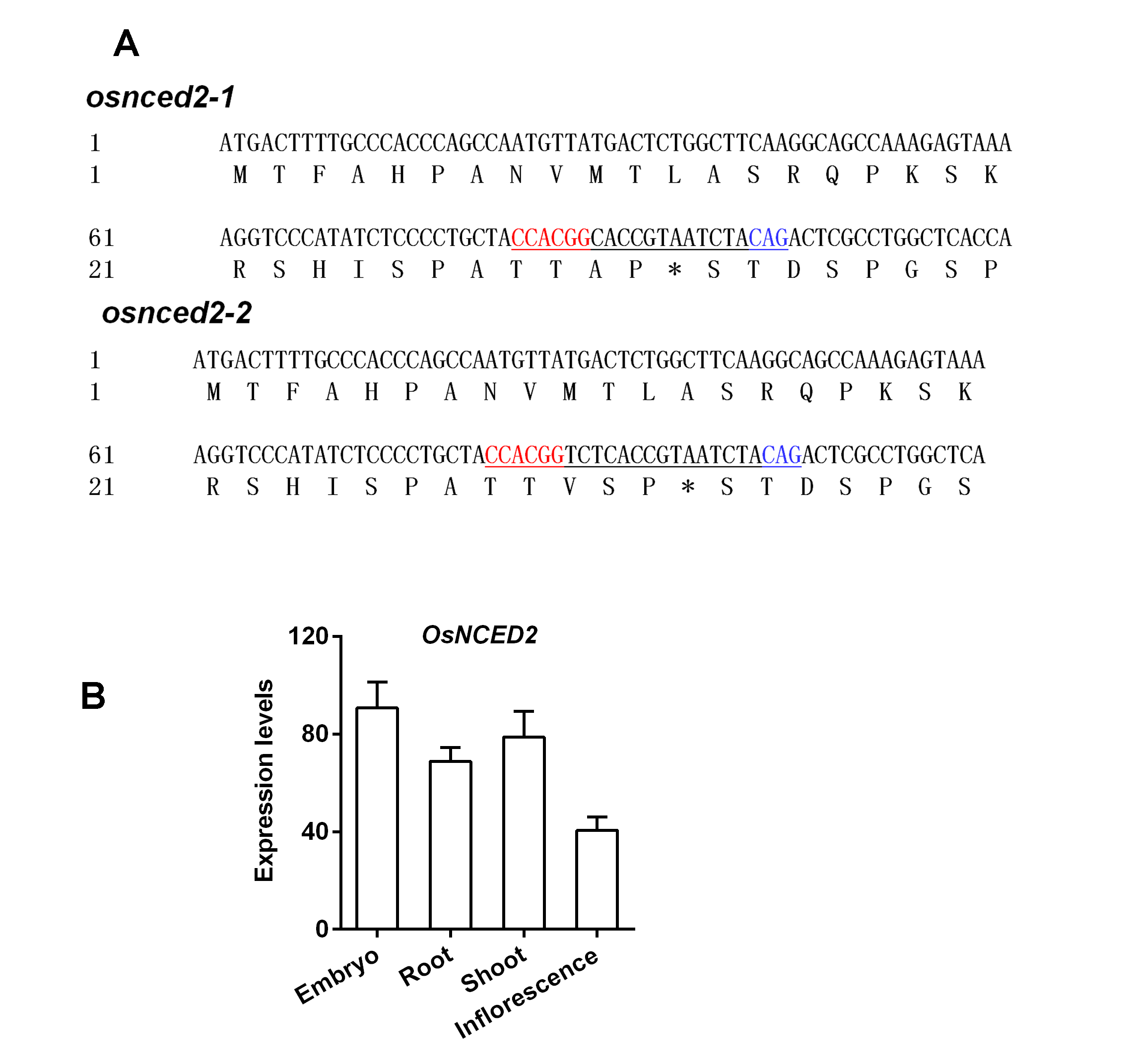

Supplement: S7 Fig — (A) Frameshift mutations of OsNCED2 leads to early termination of protein translation, resulting truncated proteins. (B) Expression profile of OsNCED2 in various tissues by quantitative PCR analysis. Error bars indicate SD with biological triplicates. (TIF) [file pgen.1009699.s007.tif]

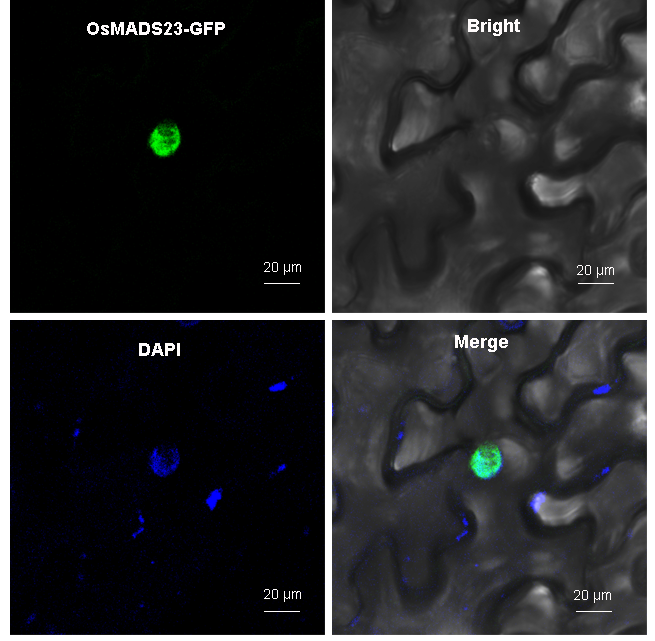

Supplement: S8 Fig — (TIF) [file pgen.1009699.s008.tif]

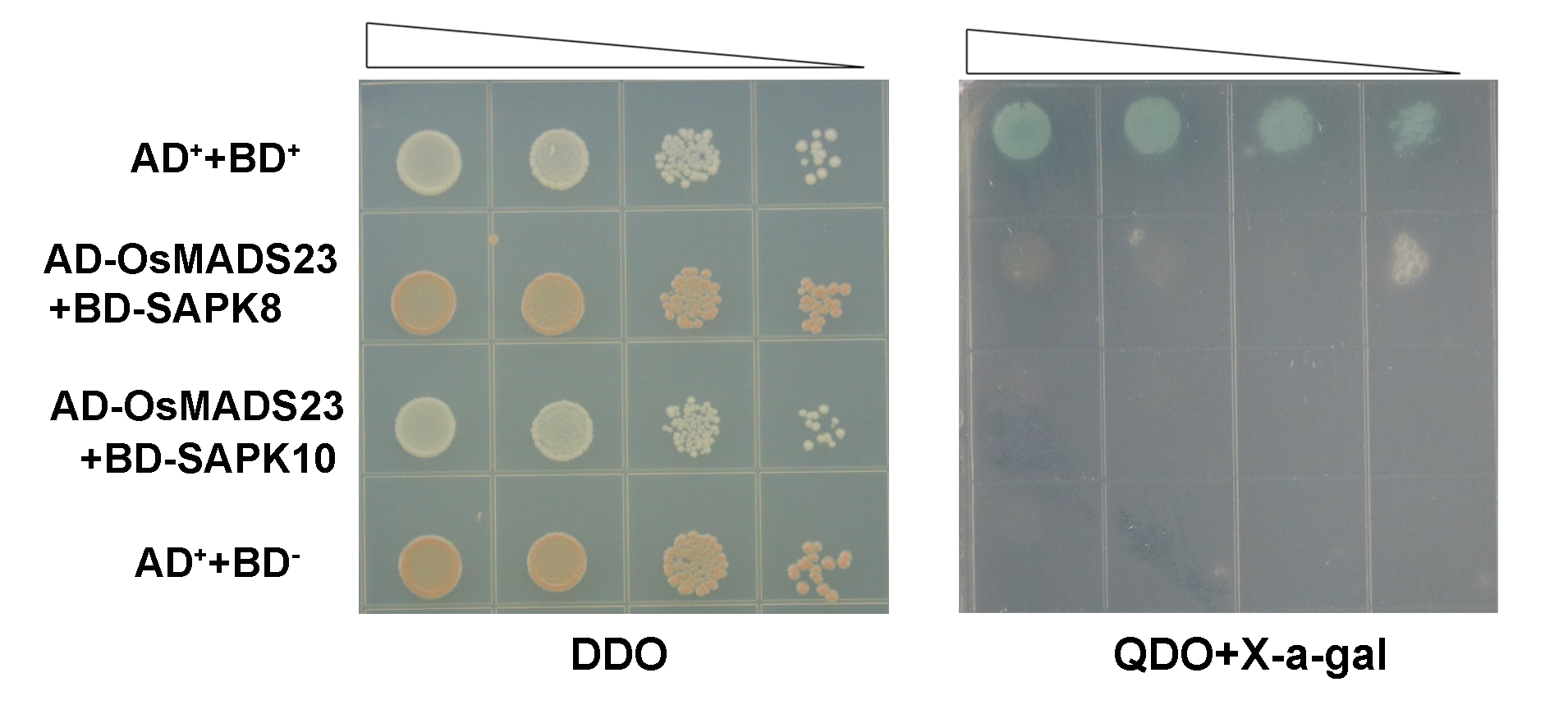

Supplement: S9 Fig — SD, synthetic dropout medium. DDO, SD/-Leu-Trp. QDO, SD/-Ade-His-Leu-Trp. (TIF) [file pgen.1009699.s009.tif]

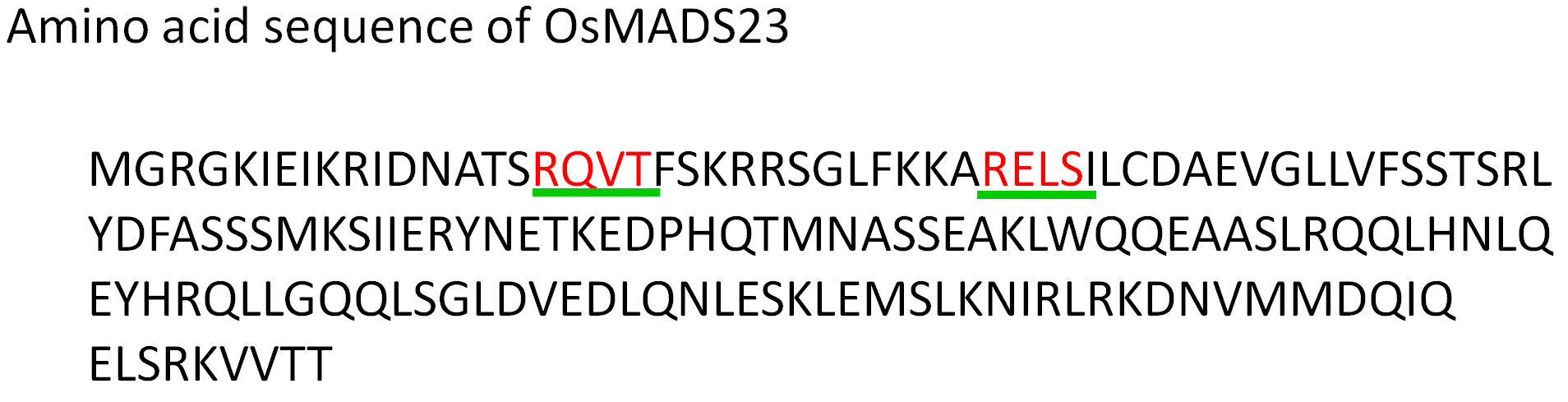

Supplement: S10 Fig — (TIF) [file pgen.1009699.s010.tif]

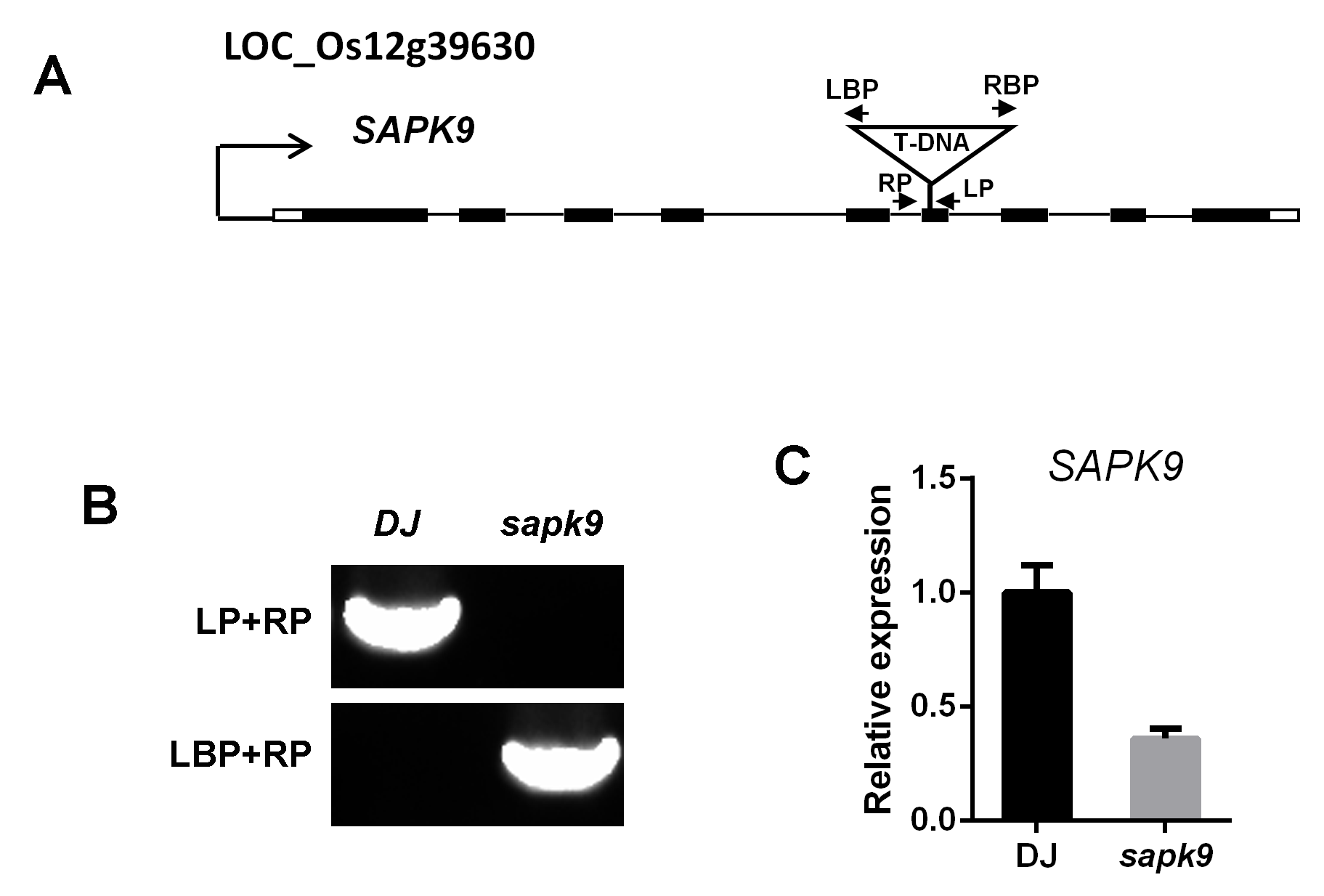

Supplement: S11 Fig — (A) Schematic diagram indicating the T-DNA insertion site in genomic region in sapk9 mutant. (B) Molecular identification of sapk9 mutant by PCR analysis. (C) Transcript levels of SAPK9 in wild type (DJ) and apk9 mutant by quantitative PCR analysis. Error bars indicate SD with biological triplicates. (TIF) [file pgen.1009699.s011.tif]

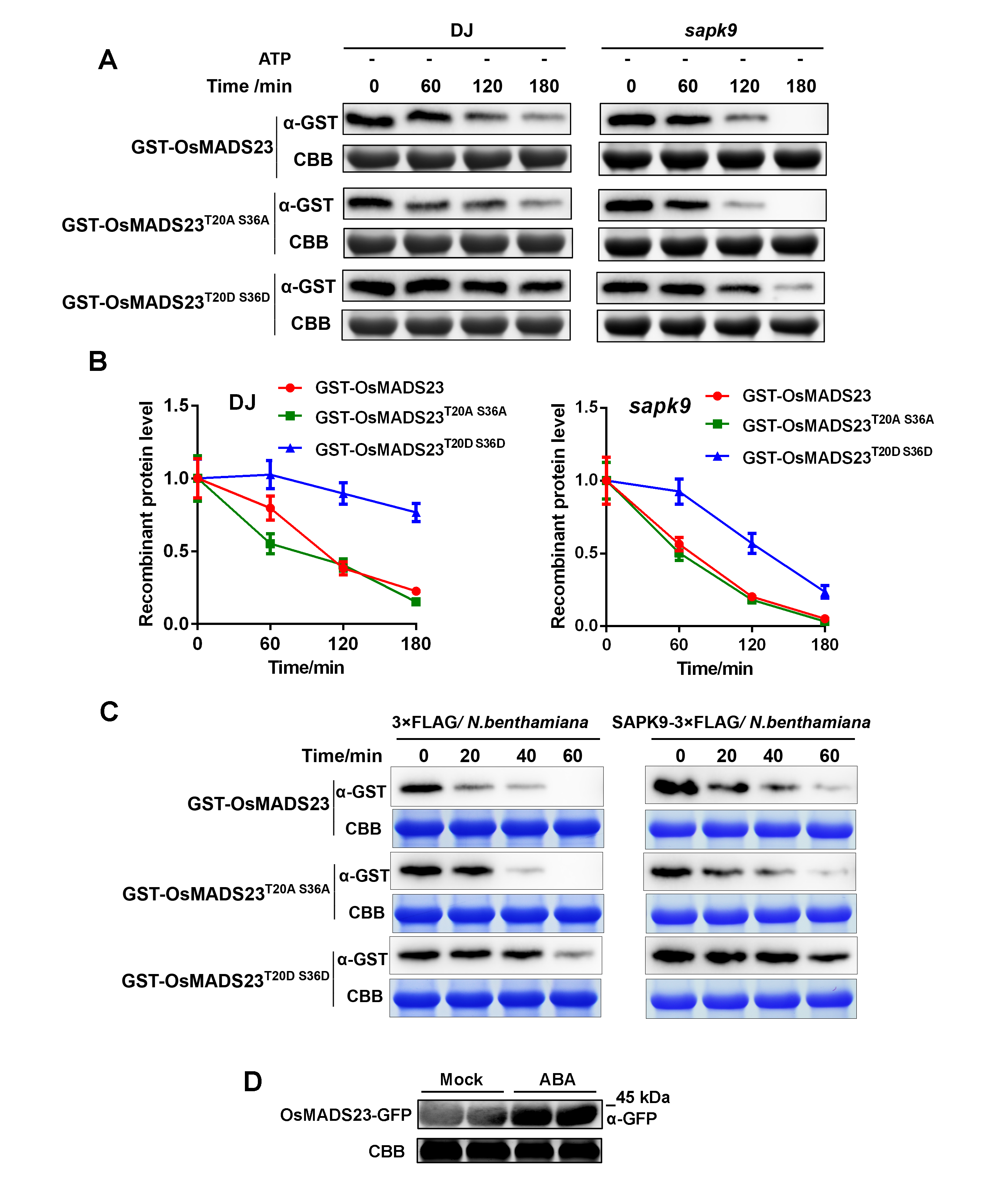

Supplement: S12 Fig — (A) The cell degradation assay of GST-OsMADS23 and its different mutated versions in the protein extracts (without ATP) from DJ (wild type) and sapk9 mutant. (B) Quantification analysis of the results described in (A). The relative levels of GST-OsMADS23 and its mutated versions in different protein extracts at 0 h were defined as 1. Data represent the means of three independent experiments. (C) The cell degradation assay of GST-OsMADS23 and its different mutated versions in the protein extracts from Nicotiana benthamiana leaves transiently overexpressing SAPK-3×FLAG or 3×FLAG alone. (D) OsMADS23 degradation in protein extracts from the OsMADS23-GFP plants treated with or without ABA. Plants were treated with 50 μM ABA for 24 h. (TIF) [file pgen.1009699.s012.tif]
