# Supplementary material for: OsMADS23 phosphorylated by SAPK9 confers drought and salt tolerance by regulating ABA biosynthesis in rice
Source: PLoS Genet. 2021 Aug 3;17(8):e1009699. doi: 10.1371/journal.pgen.1009699 (PMC8363014; doi:10.1371/journal.pgen.1009699)
Supplement: S1 Table — (DOCX) [file pgen.1009699.s013.docx]

**S1 Table Agronomic traits of *OsMADS23*-overexpressing lines and wild type (Nip)**

| Genotypes  Traits | Wild type | OE13 | OE14 |
| --- | --- | --- | --- |
| Tiller number per plant | 11.08^a^ | 15.67 ^b^ | 15.23 ^b^ |
| Effective panicle number per plant | 8.72^a^ | 12.05^b^ | 11.79 ^b^ |
| Single panicle weight (g) | 2.76^a^ | 2.08^b^ | 2.10^b^ |
| Yield per plant (g) | 24.06^a^ | 24.70^a^ | 24.76^a^ |
| 1000-grain weight (g) | 26.35^a^ | 25.86^a^ | 26.08^a^ |

Note: Plants were grown in the field for 4 months and these agronomic traits were quantified. About 300 plants were used for evaluating agronomic traits. Two-way ANOVA was performed, followed by Bonferroni’s post-hoc test**.** Different letters with the same superscript mark indicate significant differences (*p* < 0.05).
