# Supplementary material for: OsMADS23 phosphorylated by SAPK9 confers drought and salt tolerance by regulating ABA biosynthesis in rice
Source: PLoS Genet. 2021 Aug 3;17(8):e1009699. doi: 10.1371/journal.pgen.1009699 (PMC8363014; doi:10.1371/journal.pgen.1009699)
Supplement: S2 Table — (DOC) [file pgen.1009699.s014.doc]

S2 Table Primers used in this study

| **Primers for qRT-PCR** | | | |
| --- | --- | --- | --- |
| Gene Name | Primer Sequence (5’-3’) (Forward Primer/Reverse primer) | | Gene ID |
| *OsMADS23* | TGGTCCAAATCTTATTGGAGTG / TTGATGGACAAGGCTGACTTT | | [LOC_Os08g33488](http://rice.plantbiology.msu.edu/cgi-bin/ORF_infopage.cgi?orf=LOC_Os08g33488) |
| *OsCATB* | GTTCGGTTCTCCACAGTCGT / CCCTCCATGTGCCTGTAGTT | | [LOC_Os06g51150](http://rice.plantbiology.msu.edu/cgi-bin/ORF_infopage.cgi?orf=LOC_Os06g51150) |
| *OsFe-SOD* | CTTGATGCCCTGGAACCTTA / GCCAGACCCCAAAAGTGATA | | LOC_Os06g05110 |
| *OsAPX1* | CCAAGGGTTCTGACCACCTA / CAGTTCGGAGAGCTTGAGGT | | [LOC_Os03g17690](http://rice.plantbiology.msu.edu/cgi-bin/ORF_infopage.cgi?orf=LOC_Os03g17690) |
| *OsPOX1* | TTCTTCCACGACTGCTTCCC / GTGGTTGCAGAGTCAGGTTG | | [LOC_Os01g15830](http://rice.plantbiology.msu.edu/cgi-bin/ORF_infopage.cgi?orf=LOC_Os01g15830) |
| *OsP5CS1* | GTGGAGGAGGAGAGGCTG / AGTGGTGCAATACGCTACACA | | [LOC_Os05g38150](http://rice.plantbiology.msu.edu/cgi-bin/ORF_infopage.cgi?orf=LOC_Os05g38150) |
| *OsP5CR* | AATAGAGGCCATGGCTGATG / AATGCACCCTTCTCAAGCTC-3 | | [LOC_Os01g71990](http://rice.plantbiology.msu.edu/cgi-bin/ORF_infopage.cgi?orf=LOC_Os01g71990) |
| *OsHKT1* | CAACATTTGCACTGTCCAATG / AAAGACTGAGATGCAGGCAAG | | LOC_Os06g48810 |
| *OsNCED2* | GGTATGGAAACGAGGATAGTGGTT / TGCTTATTGTTGTGCGAGAAGTTC | | [LOC_Os12g24800](http://rice.plantbiology.msu.edu/cgi-bin/ORF_infopage.cgi?orf=LOC_Os12g24800) |
| *OsNCED3* | CTCACATACAGCGGCAGCAC / CGCTCGAGGACATTCGCCAC | | [LOC_Os03g44380](http://rice.plantbiology.msu.edu/cgi-bin/ORF_infopage.cgi?orf=LOC_Os03g44380) |
| *OsNCED4* | GCACGGCACCTTCATTGG / CTGTTAATTCTTTTTACTGTTGCGTTCT | | [LOC_Os07g05940](http://rice.plantbiology.msu.edu/cgi-bin/ORF_infopage.cgi?orf=LOC_Os07g05940) |
| *OsbZIP46* | GAACACTGACTGGTCCATGCTG / GAGAGAAGCAACTCTGAAGCTGAG | | [LOC_Os06g10880](http://rice.plantbiology.msu.edu/cgi-bin/ORF_infopage.cgi?orf=LOC_Os06g10880) |
| *β-actin* | AGGAAGGCTGGAAGAGGACC / CGGGAAATTGTGAGGGACAT | | [LOC_Os03g50885](http://rice.plantbiology.msu.edu/cgi-bin/ORF_infopage.cgi?orf=LOC_Os03g50885) |
| **Primers for ChIP-qPCR** | | | |
| *NCED2chip-1* | GTGGTGCTACAGTAAACATTTGC / AGCGTGTCACATCAGATATACGG | | [LOC_Os12g24800](http://rice.plantbiology.msu.edu/cgi-bin/ORF_infopage.cgi?orf=LOC_Os12g24800) |
| *NCED2chip-2* | GGGCCTTATTTACATCTTTGGGG / CCTCGTTAGATTCGTCTCGC | |  |
| *NCED2chip-3* | TCCACGTGATGTCTGTACAAT/ CGATGGGGACTCCTGTTATATT | |  |
| *NCED2chip-4* | CATCGTTGATGACCCATTATTCTC / CCCTTCAAAATGATCTGGAATG | |  |
| *NCED2chip-5* | CATGGGAAATTTGCCAAAAGG / GTGAGTACTGTGTGGACATTTGC | |  |
| *NCED3chip-1* | TTTATCTCCCCGGGGGAA / GCTTGGATTGTGGATTATGG | | [LOC_Os03g44380](http://rice.plantbiology.msu.edu/cgi-bin/ORF_infopage.cgi?orf=LOC_Os03g44380) |
| *NCED3chip-2* | ATACGGACTCGAAATCAGG / TCTTGCAAGCGGAATTTGG | |  |
| *NCED3chip-3* | CAGTACATAGAGATGCAA / GTCACATATTTTGTCCGT | |  |
| *NCED3chip-4* | CCCTTTGATCAGCATGTTA / TTGCATCTCTATGTACTG | |  |
| *NCED3chip-5* | ACTCGAACTACCACTTAGCT/ GTTTAACATGCTGATCAAAGGG | |  |
| *NCED4chip-1* | GGAGGATTCCATGCGTCT/ ATTTATAGGAGGTGGCGG | | [LOC_Os07g05940](http://rice.plantbiology.msu.edu/cgi-bin/ORF_infopage.cgi?orf=LOC_Os07g05940) |
| *NCED4chip-2* | CCCTTTACTATCAAATGCATC/ CCTTGTTGTGTGATTAACG | |  |
| *NCED4chip-3* | GAGACTATTGGATTCCTAACT/ CATTGCAACGGGTAAAATC | |  |
| *NCED4chip-4* | ATTCCCTCCCTTCGTTTCA/ TACCGGCAAAAACATCTTT | |  |
| *NCED4chip-5* | AAGTCTCCCCAATTGGTT/ CCAATACTCCTAGTAGCTT | |  |
| *OsP5CRchip-1* | CACTTATTTGTTCTTGTGCTG/ AAGCCCCAGATCCGCTGC | | [LOC_Os01g71990](http://rice.plantbiology.msu.edu/cgi-bin/ORF_infopage.cgi?orf=LOC_Os01g71990) |
| *OsP5CRchip-2* | CTCGGAAATATTCTGCCAT/ AGTGGAATTTGCGGTAGC | |  |
| *OsP5CRchip-3* | AGAACCCATTTCTGTACTAAG/ GCAAGACGTGCTTCTATTCA | |  |
| *OsP5CRchip-4* | GTGCTGGGTCTCCAATGTAT/ GATCATGAAATCATCGCACA | |  |
| **Primers for EMSA assay** | | | |
| *OsNCED2-a –*biotin | AACATTCTATCCCATAAAATTGTGTTTTGTTGCAAATGTCC / GGACATTTGCAACAAAACACAATTTTATGGGATAGAATGTT | | [LOC_Os12g24800](http://rice.plantbiology.msu.edu/cgi-bin/ORF_infopage.cgi?orf=LOC_Os12g24800) |
| *OsNCED2-b –*biotin | GGGGTTGTCTATGTAACATATAATTGGATATATATTCATTCC /  GGAATGAATATATATCCAATTATATGTTACATAGACAACCCC | | [LOC_Os12g24800](http://rice.plantbiology.msu.edu/cgi-bin/ORF_infopage.cgi?orf=LOC_Os12g24800) |
| *OsNCED2-c–*biotin | GGGGCTAATAATTAGCCTTCCAAAATTTTGCTATTTATAAGTA / TACTTATAAATAGCAAAATTTTGGAAGGCTAATTATTAGCCCC | | [LOC_Os12g24800](http://rice.plantbiology.msu.edu/cgi-bin/ORF_infopage.cgi?orf=LOC_Os12g24800) |
| **Primers for generating DNA vectors (The underline showed the restriction enzyme sites)** | | | |
| Gene name (ID) | Primer Sequence (5’-3’) (Forward Primer/Reverse primer) | Bone vector | Experimental purpose |
| *OsMADS23*  ([LOC_Os08g33488](http://rice.plantbiology.msu.edu/cgi-bin/ORF_infopage.cgi?orf=LOC_Os08g33488)) | gtgaGGTACCATGGGGAGAGGGAAGATAGAG /  gtgaGGATCCAGAACCACCACCAGAACCACCACCAGTGGTAACAACCTTCCTGC | pCAMBIA-1301 | *OsMADS23* overexpression vector |
|  | gtgaGGTACCATGGGGAGAGGGAAGATAGAG / gtgaGGATCCAGAACCACCACCAGAACCACCACCAGTGGTAACAACCTTCCTGC | pCAMBIA-1301-GFP | OsMADS23-GFP fusion protein |
|  | gtgaGGATCCATGGGGAGAGGGAAGATAGAG / gtgaCTCGAGCTAAGTGGTAACAACCTTCCTGC | pGreenII 62-SK | Transient transactivation assay |
|  | gagtGAATTC ATGGGGAGAGGGAAGATAGAG /  gagtCTCGAG AGTGGTAACAACCTTCCTGC | PGADT7 | Yeast two-hybrid assay |
|  | gagtTCTAGA ATGGGGAGAGGGAAGATAGAG /  gagtGGATCC AGTGGTAACAACCTTCCTGC | PAB855 | BIFC |
|  | gagtGAATTC ATGGGGAGAGGGAAGATAGAG /  gagtCTCGAG CTAAGTGGTAACAACCTTCCTGC | pCold TF | Recombinant His-OsMADS23 |
|  | gagtGAATTC ATGGGGAGAGGGAAGATAGAG /  gagtCTCGAG CTAAGTGGTAACAACCTTCCTGC | pGEX-4T-1 | Recombinant  GST-OsMADS23 |
|  | gagtGAATTCATGGGGAGAGGGAAGATAGAGATAAAGAGGATCGACAACGCGACGAGCCGACAGGTGGCATTCTC /  GATGGCGAGCTCCCTCGC | pGEX-4T-1 | Recombinant  GST-OsMADS23 ^T201A S36A^ |
|  | GCGAGGGAGCTCGCCATC /  gagtCTCGAGCTAAGTGGTAACAACCTTCCTGC |  |  |
|  | gagtGAATTCATGGGGAGAGGGAAGATAGAGATAAAGAGGATCGACAACGCGACGAGCCGACAGGTGGCATTCTC/ gagtCTCGAGCTAAGTGGTAACAACCTTCCTGC |  |  |
|  | gagtGAATTCATGGGGAGAGGGAAGATAGAGATAAAGAGGATCGACAACGCGACGAGCCGACAGGTGGACTTCTC/ GATGTCGAGCTCCCTCGC | pGEX-4T-1 | Recombinant  GST-OsMADS23 ^T201D S36D^ |
|  | GCGAGGGAGCTCGACATC /  gagtCTCGAGCTAAGTGGTAACAACCTTCCTGC |  |  |
|  | gagtGAATTCATGGGGAGAGGGAAGATAGAGATAAAGAGGATCGACAACGCGACGAGCCGACAGGTGGACTTCTC / gagtCTCGAGCTAAGTGGTAACAACCTTCCTGC |  |  |
| *SAPK9*  ([LOC_Os12g39630](http://rice.plantbiology.msu.edu/cgi-bin/ORF_infopage.cgi?orf=LOC_Os12g39630)) | gagtTCTAGAATGGAGAGGGCGGCGGCGG /  gagtGTCGACCATGGCATATACGATCTCTCCGC | pCAMBIA-1301 | Fusion protein  SAPK9-3**×**FLAG |
|  | gagtGGTACCATGGAGAGGGCGGCGGCGG  TCCAACTGTTGCTTTTGGTTG  CAACCAAAAGCAACAGTTGGA  gagtTCTAGACATGGCATATACGATCTCTCCGC |  | Fusion protein SAPK9^S176A^-3× FLAG |
|  | GAATTC ATGGAGAGGGCGGCGGCGG /  CTCGAG CATGGCATATACGATCTCTCCGC | pET32a | Recombinant  His-SAPK9 |
|  | gagtGAATTCATGGAGAGGGCGGCGGCGG  TCCAACTGTTGCTTTTGGTTG  CAACCAAAAGCAACAGTTGGA  gagtCTCGAGCATGGCATATACGATCTCTCCGC | pET28a | Recombinant  His-SAPK9^S176A^ |
|  | gagtGAATTCATGGAGAGGGCGGCGGCGG /  gagtCTCGAGTTACATGGCATATACGATCTCTCCGC | pGEX-4T-1 | Recombinant  GST-SAPK9 |
|  | gagtCATATGTTTGCGTTTGTGTTCGTT /  gagtGAATTCTTACATGGCATATACGATCTCTC | PGBKT7 | Yeast two-hybrid assay |
|  | gagtAGATCTATGGAGAGGGCGGCGGC /  gagtTCTAGACATGGCATATACGATCTCTCCGC | PAB862 | BIFC |
| *SAPK8*  ([LOC_Os03g55600](http://rice.plantbiology.msu.edu/cgi-bin/ORF_infopage.cgi?orf=LOC_Os03g55600)) | gagtCATATGCCTTGGAAGCCGCAGGTAGGTAGAAC /  gagtGCGGCCGCTTACATCGCATAGACGATCTCGCCGC | PGBKT7 | Yeast two-hybrid assay |
| SAPK10  ([LOC_Os03g41460](http://rice.plantbiology.msu.edu/cgi-bin/ORF_infopage.cgi?orf=LOC_Os03g41460)) | gagtCATATGGCGAGAGCTTCATTCACGGGAGGTTG /  gagtGCGGCCGCTCACATAGCGTATACTATCTCCCCACTGCTTTCC | PGBKT7 | Yeast two-hybrid assay |
| *OsNCED2*  ([LOC_Os12g24800](http://rice.plantbiology.msu.edu/cgi-bin/ORF_infopage.cgi?orf=LOC_Os12g24800)) | CTCGAGCGTGTGTGCAATCTAAATCCG / CTGCAGCCTCGTTAGATTCGTCTCGC | pGreenII 0800 | Transient transactivation assay(LUC) |
|  | CTCGAGCGTGTGTGCAATCTAAATCCG / CTGCAGCCTCGTTAGATTCGTCTCGC | pGreenII 0800-GUS | Transient transactivation assay (GUS) |
| *OsNCED3*  ([LOC_Os03g44380](http://rice.plantbiology.msu.edu/cgi-bin/ORF_infopage.cgi?orf=LOC_Os03g44380)) | CTCGAGCTTCACTAAAATTAGACCATAA / GATATCTATACGGTTGGTTCCTAT | pGreenII 0800 | Transient transactivation assay |
| *OsNCED4*  ([LOC_Os07g05940](http://rice.plantbiology.msu.edu/cgi-bin/ORF_infopage.cgi?orf=LOC_Os07g05940)) | CTCGAGATACCCAGATTTCCATACATT / GATATCCCTATCAAATTAAACCAGTA | pGreenII 0800 | Transient transactivation assay (LUC) |
| *OsP5CR*  ([LOC_Os01g71990](http://rice.plantbiology.msu.edu/cgi-bin/ORF_infopage.cgi?orf=LOC_Os01g71990)) | CTCGAGAGTACGGTAGTAGCATCCAA / GATATCTGCGATAAGAACAGGCTCCC | pGreenII 0800 | Transient transactivation assay (LUC) |
| **Primers for identifying *sapk9* knockout mutant** | | | |
| GCAGTCATCGGTTCTTCATT/GGTTCTTTATCTCAGGCATGG  GCAGTCATCGGTTCTTCATT/CTAGAGTCGAGAATTCAGTACA | | | |
| **Primers for identifying *osmads23* knockout mutants** | | | |
| ACCCTTAGGACATGCTTTGC/ TGATCCATCATGACATTGTCC  ACCCTTAGGACATGCTTTGC/ TGCAGGTTCTCTCCAAAT | | | |
| **Primers for identifying *osnced2* CRISPR/CAS9 mutants** | | | |
| AGCCTTACTCTGGCGATCTATATATAG / GTGCACATAGCGTGAACAAAGCA | | | |
